# Wireless, Adaptable and Fully Implantable Battery‐powered Devices for Optical Stimulation of the Spinal Cord in Small Rodents

**DOI:** 10.1002/advs.75419

**Published:** 2026-04-22

**Authors:** Shahriar Shalileh, Adan Moallemi, Elham Mohseni Vadeghani, Samuel Tretjakov, Brianna Tsuyuki, Alexander Golab, Benjamin Rever, Wolfram Tetzlaff, Dena Shahriari

**Affiliations:** ^1^ School of Biomedical Engineering University of British Columbia (UBC) Vancouver British Columbia Canada; ^2^ International Collaborations on Repair Discoveries (ICORD) Vancouver British Columbia Canada; ^3^ Department of Zoology University of British Columbia Vancouver British Columbia Canada; ^4^ Department of Orthopaedics University of British Columbia Vancouver British Columbia Canada

## Abstract

Existing technologies for optogenetic neuromodulation often rely on external power transmission or are only partially implantable, limiting their utility in mobile tissues such as the spinal cord. Here, we introduce a fully implantable, battery‐powered system for light delivery to precise segments of the spinal cord in freely behaving small rodents, with operation and wireless recharging possible in any animal housing environments. Built from commercially available components with minimal in‐house fabrication, the system integrates low‐power electronic design, ultra‐thin and flexible probes with multiple embedded microscale light‐emitting diodes (µLEDs), coupled to a rechargeable battery in a modular configuration. The adaptable device can be used for optical stimulation with different wavelengths across various spinal levels with minimal system adjustments. Month‐long stability as well as the device functionality were validated in small rodents. Furthermore, operator‐free, targeted and transient paralysis of the hind limb was demonstrated through controlled optical stimulation of inhibitory interneurons.

## Graphical abstract

Current technologies for spinal cord optogenetic stimulation rely on external power sources and face reliability constraints in freely behaving animals. Here, a fully implantable, battery‐powered optoelectronic device is introduced, enabling operation in any selected environment with wireless recharging for months‐long stimulation. We show reversible hindlimb paralysis by targeted activation of inhibitory spinal interneurons, demonstrating segment‐specific neuromodulation and device adaptability.

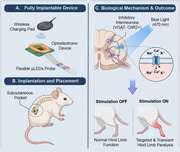

## 1 Introduction

By combining genetic modifications and light‐delivery modalities, optogenetics has enabled cellular‐precision modulation accuracy and control [1, 2, 3, 4]. Optogenetics is widely used to investigate underlying physiological mechanisms, particularly in the brain [5], which has typically been achieved by the insertion and fixation of optical fibers to the skull for light delivery [6, 7]. However, the rigidity of fiber optics prohibits their implantation in relatively more mobile and flexible tissues such as the spinal cord [8]. Alternatively, soft, flexible µLED‐carriers have been positioned epidurally, adjacent to the spinal cord dura matter [9, 10]. These technologies include headstaged and backpacked platforms, which enable access to the implanted components to power the system, control the optical delivery parameters and wirelessly transmit/receive data. However, these platforms can affect natural animal behavior, especially in studies of social behavior [11]. In addition, the connection between the implanted components and the external platform might get faulty or cause infection over time, impeding long‐term studies [12, 13]. Fully implantable, battery‐free devices have significantly reduced chances of external infection or damage to the dermal tissue [11, 14, 15]. However, the main limitation of current battery‐free technologies is with the wireless radiofrequency (RF) power transmission, which requires animal placement in custom cages to receive the energy required for optical stimulation. In addition, the wireless power transfer to the implant is influenced by both the spatial position of the animal within the cage and the geometric configuration of the power transmitter and can vary by up to 50% [16, 17]. Supplementary Table S1 highlights current battery‐ free modalities along with the advantages and disadvantage of the technology.

Here, we introduce a simple‐to‐reproduce, fully implantable and wirelessly rechargeable battery‐powered optoelectronic system to deliver optical stimulation to specific segments of the spinal cord in both mouse and rat animal models. We developed modular µLED probes that interface with a common electronic circuit, enabling adaptation to diverse experimental applications without redesigning the entire device. To reduce the overall device size and address the common limitation of battery‐powered systems, which typically require substantial implantation space and are often in tethered configurations [15, 18], we designed a fully implantable architecture that spatially separates the battery and the electronic core. This separation is enabled by soft, stretchable interconnections that distribute device volume and mass across anatomically suitable implantation sites. The use of industry‐compatible manufacturing and commercially available components eliminates the need for special clean room equipment, providing an accessible toolkit for optical modulation. Furthermore, we have developed a low‐cost battery‐charging unit that is compatible with widely available wireless chargers. This fully implantable and wirelessly rechargeable light‐delivery system enables month‐long behavioral studies in diverse environments, such as the horizontal ladder task, CatWalk, or large open field test (OFT), which are cumbersome or even impossible to conduct using current technologies.

## 2 Results

### 2.1 Flexible Micro‐LED Probe Design

**FIGURE 1:**
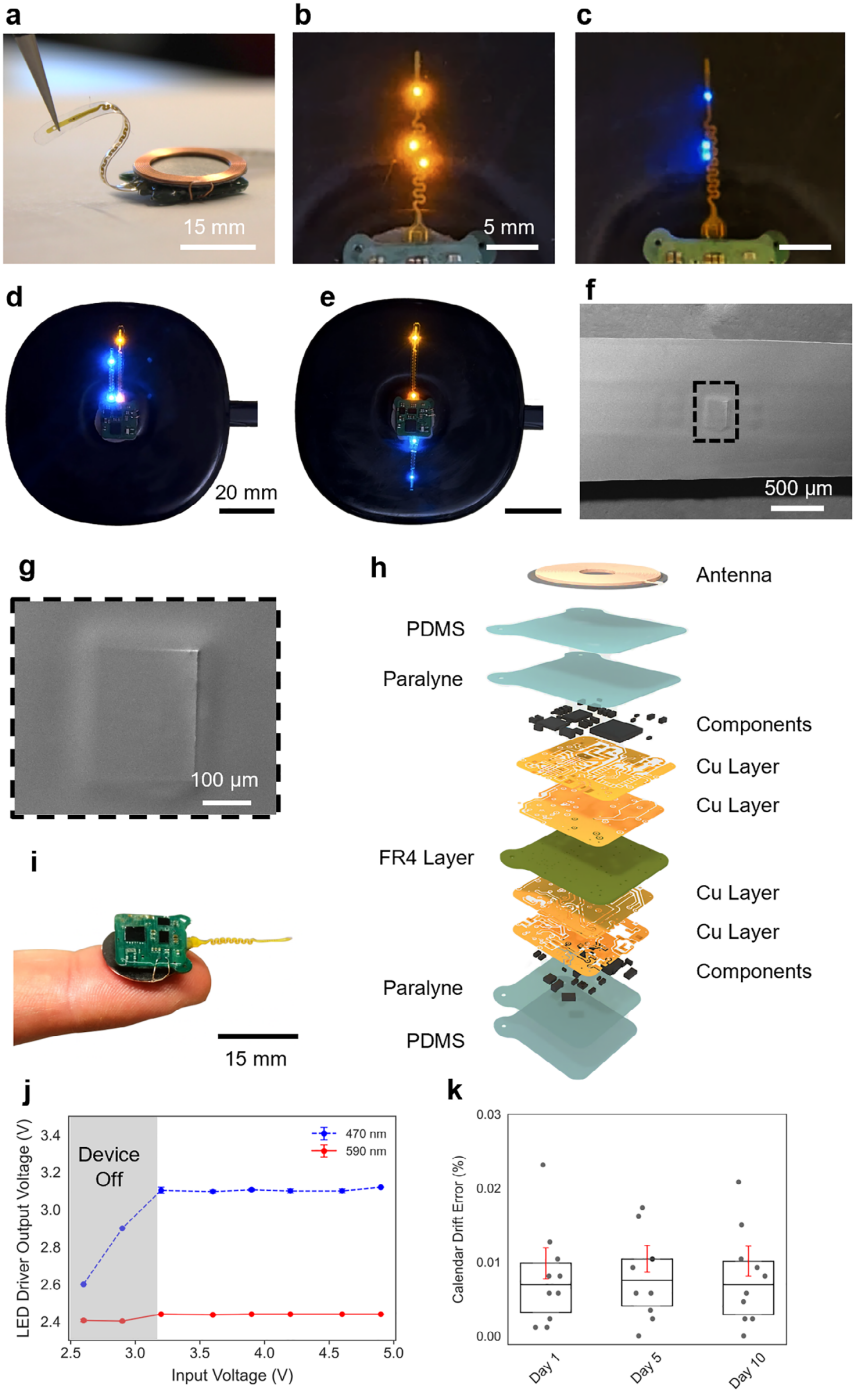
The fully implantable, battery‐powered optoelectronic device. (a) The flexible micro‐LED probe connected to the optoelectronic device. (b,c) The probes with laser‐ablated openings in the soldermask for defined µLEDs placement along the probe for amber (b) and blue (c) lights. (d,e) Photographs of the device driving two µLED probes with independent optical parameters and different µLEDs on each probe. (f,g) Top view SEM image of a fully encapsulated probe (f) and zoomed‐in view of the encapsulated µLED (g). (h) Exploded‐view schematic of the electronic core illustrating the layered architecture, including the receiver coil, encapsulation layers and control circuitry. (i) The device resting on a fingertip. (j) Electrical output voltage of the LED driver as a function of input voltage (*n* = 8, mean ± s.e.m.). The device shuts down for input voltages below 3.1 V. (k) Temporal drift error of the calendar over 10 days of continuous operation (*n* = 10, mean ± s.e.m.).

By utilizing flexible printed circuit board (fPCB) technology, we designed polyimide/copper/polyimide (PI/Cu/PI) ultrathin (∼40 µm) µLED probes (Figure 1a). Multiple µLEDs can be integrated at designated positions on the substrate, enabling localized stimulation at targeted levels along the spinal cord. The precise µLED placement was facilitated by laser ablation of the second polyimide (PI) layer to selectively expose solder contact pads (Figure 1b,c). Such an approach enabled high‐yield, manual assembly without reliance on automated pick‐and‐place machines, which are often expensive to use and generally not readily available at laboratory scale [19]. In addition, the µLED probes were fabricated in various lengths, widths and different µLEDs configurations to accommodate differences in local spinal cord anatomy, anatomical variability across species (mice and rats), as well as wavelength‐specific stimulation needed to activate different opsins (Figure 1d,e and Extended Data Figure S1a,b). To ensure electrical hermiticity and mechanical conformity, the µLED probes were encapsulated in a dual‐layer coating comprising a thin Parylene C (∼5 µm) and a PDMS layer (∼70 µm) [10, 20]. The structure conforms to the soft, curved anatomy of the spinal cord while minimizing optical scattering before reaching the dura mater [21]. Scanning Electron Microscopy (SEM) confirmed the uniformity of encapsulation along the µLED probe and full coverage of the µLEDs (Figure 1f,g and Extended Data Figure S1c,d).

### 2.2 Adaptive Design of the Programmable Electronic Core

We aimed to develop a miniaturized electronic core suitable for chronic subdermal implantation, capable of driving multiple µLED probes with distinct stimulation parameters. We designed an electronic platform (0.9 g, 0.3 cm^3^) featuring an ultra‐low power microcontroller unit (MCU) to enable programmable optical stimulation (Figure 1h,i and Extended Data Figure S2a). Two independent LED drivers deliver steady, constant currents to µLEDs by dynamically adjusting the variable input voltage. Each driver can be configured to control a distinct set of µLEDs with a broad range of electrical parameters (current 1–300 mA, frequency 0.1–200 Hz), enabling the use of different µLEDs without requiring any hardware or software modifications (Supplementary Video S1). Particularly, the electrical output of the LED drivers, programmed to deliver constant current of 20 mW to µLEDs with two different wavelengths (470 nm and 590 nm), remained stable across a varying range of battery voltage levels and during active wireless charging (Figure 1j and Extended Data Figure S2b). This dual‐channel architecture supports spatially independent, multimodal neurostimulation, with full control over wavelength, pulse width, frequency, duty cycle, and irradiance profile (Supplementary Video S2 and S3).

To enable reliable month‐long studies with precise stimulation control, we utilized the calendar module of the MCU in combination with an external piezoelectric crystal oscillator to generate stable, drift‐resistant time steps. This configuration minimized the temporal drift of the MCU internal oscillator, which can be as high as ∼10% under physiologically relevant thermal and humidity conditions [22] to less than 0.01% (Figure 1k and Extended Data Figure S2c). These features offer autonomous, month‐long neuromodulation experiments without daily user intervention. The final assembled optoelectronic device was encapsulated in a dual‐layer conformal coating consisting of ∼12 µm of Parylene‐C and an outer PDMS layer (400–600 µm), forming an insulative interface suitable for month‐long implantation [11, 16].

### 2.3 Device Powering by an Implantable Battery Connected via Soft and Flexible Interconnections

**FIGURE 2:**
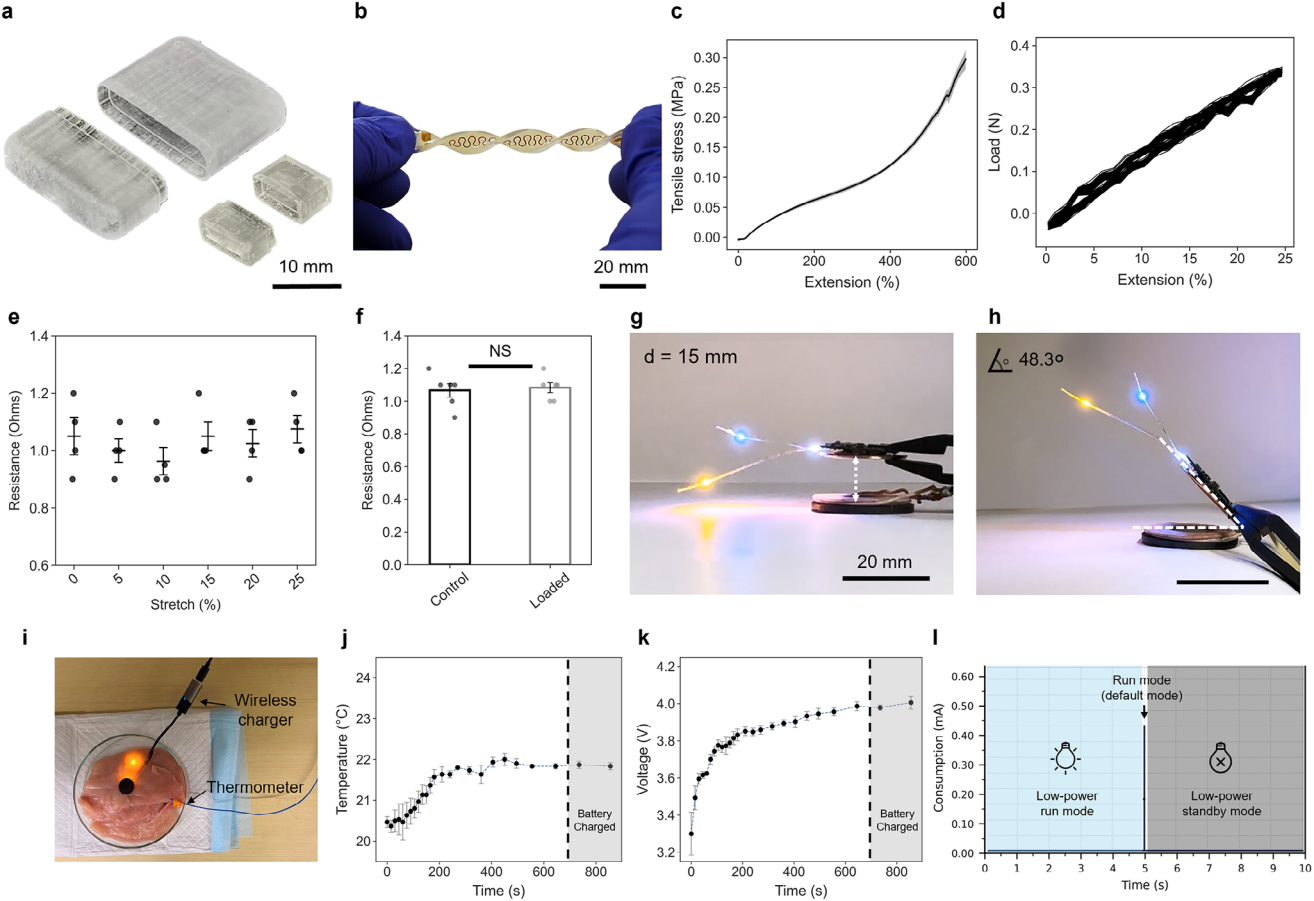
Characterization of the battery, soft interconnection and wireless charging unit. (a) Custom 3D‐printed medical‐grade photopolymer cases housing Li‐Po batteries of different sizes used in rats (left) and mice (right) implantations. (b) Fabricated interconnection under manual stretching. (c) Stress–strain curve via uniaxial tensile testing showing yield strain of >300% and ultimate tensile strength at ∼600% strain (*n* = 5). (d) Cyclic loading‐unloading curves at 25% strain for 20 000 cycles at 1 Hz (*n* = 4). (e) Electrical resistance of the soft interconnections under tensile strain from 0%–25% (n = 4, mean ± s.e.m.). (f) Electrical resistance before and after cyclic loading (*n* = 6, paired Student's *t*‐test: p = 0.75, mean ± s.e.m.). (g,h) Wireless charging tolerance to lateral coil displacement (g) and angular misalignment (h). (i) Photograph of the thermal measurement setup. The device is placed in a chicken breast and being wirelessly charged, while a thermometer probe measuring the device platform temperature. (j) Device platform temperature–time profile during wireless charging with modified receiver antenna shielding (*n* = 3, mean ± s.e.m.). (k) Battery voltage–time profile during a full wireless charging cycle of a battery (150mAh). A full charging cycle is completed in approximately 14 min (*n* = 3, mean ± s.e.m.). (l) Power consumption profile of the device, depicting the low‐power run mode (optical stimulation) and the transition to the low‐power standby mode (no optical stimulation). The mode transition occurs within 2.2 ms (Default run mode).

Lithium‐polymer (Li‐Po) batteries are efficient and reliable power sources primarily because of their high energy density and relatively stable discharge voltage [23]. However, their safe implantation remains an important consideration in biomedical devices since they pose potential risks such as cytotoxic byproducts and electrolyte leakage for chronic use [15]. To address these considerations, we developed custom encapsulations as protective cases using stereolithography (SLA) 3D printing with a medical‐grade photopolymer. This approach provides a scalable, cost‐effective, and reproducible method for rapid prototyping and consistent production. The cases can be dimensionally optimized to fit various battery sizes, enabling flexible integration of different power capacities depending on the optical stimulation energy required for different applications (Figure 2a and Extended Data Figure S2d).

To ensure a reliable electrical pathway and stable voltage delivery to the circuit in vivo, we developed soft, flexible interconnections that maintained continuous connectivity between the battery and the optoelectronic device while accommodating dynamic muscle movement along the body (Figure 2b). Extended Data Figure S2e illustrates the schematic structure of the soft interconnections. A double‐layer fPCB was encapsulated between two layers of EcoFlex, forming a flexible and stretchable interface between the battery and the electronic core. To assess mechanical robustness, we performed uniaxial tensile testing on the interconnections. The Young's modulus was measured to be 54 ± 8 KPa (Figure 2c), which was previously suggested to be in a range suitable for skin and subcutaneous muscle tissues [24, 25]. The material also exhibited a yield strength of about 100 KPa at > 300% strain. To evaluate fatigue resistance under month‐long use, we conducted a cyclic loading–unloading test up to 25% strain (20 000 cycles, 1 Hz), which surpasses the maximum strain levels reported during natural rodent locomotion [10, 26] (Figure 2d). Hysteresis and subsequently plastic deformation were not observed. Live measurements showed that the electrical resistance of the interconnections remained stable across different stretch levels (Figure 2e). Notably, after 20 000 loading cycles, the resistance was reassessed and found to be unchanged (Figure 2f).

### 2.4 Fast Wireless Charging of the Optoelectronic Device

We designed and implemented a custom Qi‐compatible power receiver for wireless battery charging, leveraging Qi as an inductive charging standard widely used in consumer electronics but not yet broadly adopted in biomedical devices [27]. Specifically, since the Qi interface operates in the low frequency range (110–205 kHz), it enables safe energy transmission through biological tissue without inducing significant dielectric heating [28]. Furthermore, this technology enables compatibility with commercially available transmitters. To assess the geometric misalignment sensitivity between the charging coil (transmitter) and the receiver antenna embedded in the implant, we conducted a controlled bench test. Reliable voltage buildup at the receiver was observed at lateral displacements reaching 15 mm (Figure 2g and Extended Data Figure S2f) and angular offsets of up to 48° (Figure 2h and Extended Data Figure S2g), demonstrating a broad alignment tolerance suitable for reliable battery charging. Initially, the commercially acquired receiver antenna was equipped with a built‐in ferrite shielding layer to enhance the magnetic influx and thus reduce thermal dissipation. However, during our pre‐implantation bench testing, the device exhibited heating exceeding 80°C under minimal load conditions (without a connected battery). Thermal analysis of the device when charging a battery showed a rapid increase in temperature, exceeding 90°C in less than 60 s (Extended Data Figure S2h), which compromises temperature‐sensitive components such as the embedded crystal quartz [22]. The temperature rise was most pronounced near the power management circuitry, which contains a high density of copper traces and planes and was attributed to the absorption of stray magnetic fields by the surrounding metallic objects [29]. To reduce the temperature rise, we laser cut an additional ferrite shielding layer and placed it under the receiver coil to provide additional electromagnetic shielding. Thermal characterization was further performed under a physiologically relevant condition by placing the device within an ex vivo tissue (chicken breast) during wireless charging. The device exhibited a maximum temperature increase of approximately 1.7°C under tissue‐embedded conditions (Figure 2i,j), remaining well within safe operating limits throughout the charging cycle. Notably, the temperature rise for the battery or the soft interconnection was not detected. The battery voltage–time profile during a complete wireless charging cycle is shown in Figure 2k. The voltage profile showed a non‐linear rise common with Li‐Po batteries, with a steady increase during the constant‐current phase of charging. A full charge of a 150 mAh LiPo battery was completed in ∼14 min, supporting continuous operation of a single blue µLED (470 nm) for more than 90 h at 20 Hz with 10% duty cycle. Supplementary Videos S4 and S5 show wireless charging of implanted devices in anesthetized as well as awake rats, with free handling of them, respectively. Charging was performed using a commercially available charger equipped with an LED indicator that illuminates during real‐time power transmission, with transmitter–receiver coil alignment facilitated by a small magnet attached to the transmitter (Extended Data Figure S3 and Supplementary Note S1). Different programmable power modes of the MCU, corresponding to various device states, together with their associated power consumption profile, are presented in Extended Data Figure S4a. Such ultra‐low power consumption (Figure 2l) and rather short charging period lower the frequency and period of animal restraint to recharge the implanted battery.

### 2.5 Computational Modelling of Optical Penetration and Associated Thermal Effect

**FIGURE 3:**
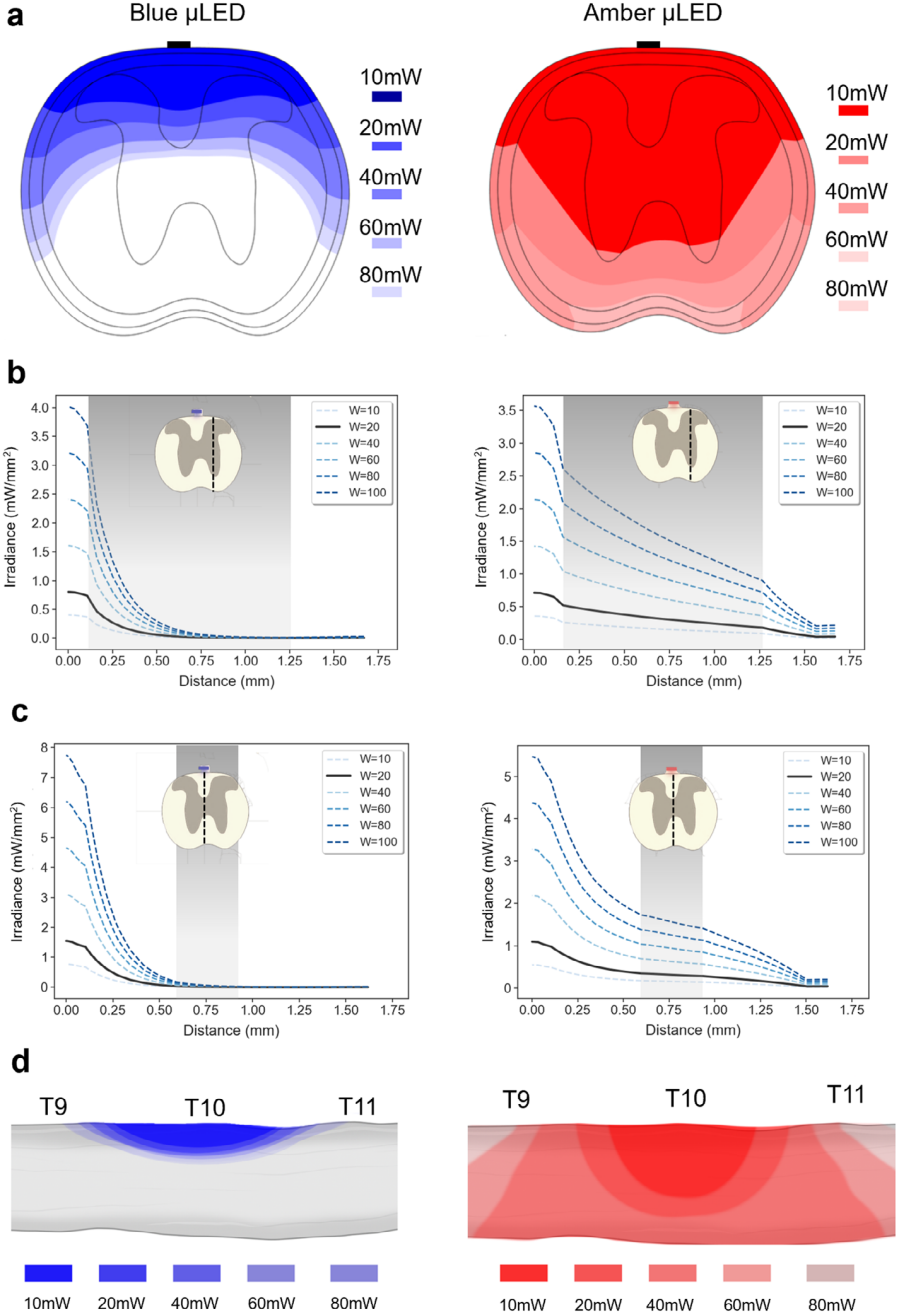
Optical characterization of the light‐tissue interaction in mice. (a) Simulated optical irradiance distributions for blue, 470 nm (left) and amber, 590 nm, (right) µLEDs in a mouse spinal cord for electrical input power ranging from 10 to 80 mW. Regions with magnitudes below the threshold of 0.1 mW/mm^2^ were excluded from the simulation results. (b,c) Irradiance profiles along the dorsal–ventral axis for blue (left) and amber (right) light, shown for two distinct cut lines. Grey shading marks the depth range corresponding to the spinal cord gray matter relative to the µLED. (d) Side‐view optical intensity profile for a blue (left) and an amber (right) µLED positioned at T10.

To evaluate light‐tissue interaction within the spinal cord, including light irradiance distribution, penetration depth, and thermal effects, we performed simulations via finite element analysis (FEA) in mouse and rat spinal cord models. As shown in Extended Data Figure S5a, we considered distinct anatomical layers, gray matter, white matter, cerebrospinal fluid (CSF), and epidural fat in the computational model to realistically represent the tissue environment in vivo. To evaluate the impact of wavelength variation on propagation characteristics, we compared the light irradiance distribution produced by blue and amber µLEDs (470 and 590 nm, respectively), as two common wavelengths used in optogenetic studies [30]. Figure 3a shows the optical intensity distribution across a range of electrical input powers (10–80 mW) in the mouse spinal cord. Quantitative profiles of light intensity along dorsal‐to‐ventral paths demonstrated that blue light intensity dropped sharply near the surface, whereas amber light exhibited a more gradual decline, allowing deeper penetration into the ventral region (Figure 3b,c). This difference is attributed to the higher absorption and scattering of blue light in white matter [31], where it rapidly dissipates near the spinal cord surface. Figure 3d shows the side‐view light intensity profile for a µLED positioned at T10. In contrast to the narrow, focused illumination pattern of a laser‐based delivery, the µLED produced a broader spatial distribution, spanning about three spinal segments (T9‐T11). This wider emission angle of µLEDs can be used in applications where larger areas are desired, for example, dorsal horn stimulation, while stimulations via laser offer more precise, narrower targeting of the cells in a specific spinal cord region. We then repeated the simulations in a rat spinal cord model to account for interspecies anatomical differences. Blue light penetration in the rat model was limited, hardly reaching regions located in the dorsal horn (Extended Data Figure S6). This was primarily due to the increased thickness of the white matter and the larger dimensions of the spinal cord in rats, which increased the optical path length and associated scattering.

**FIGURE 4:**
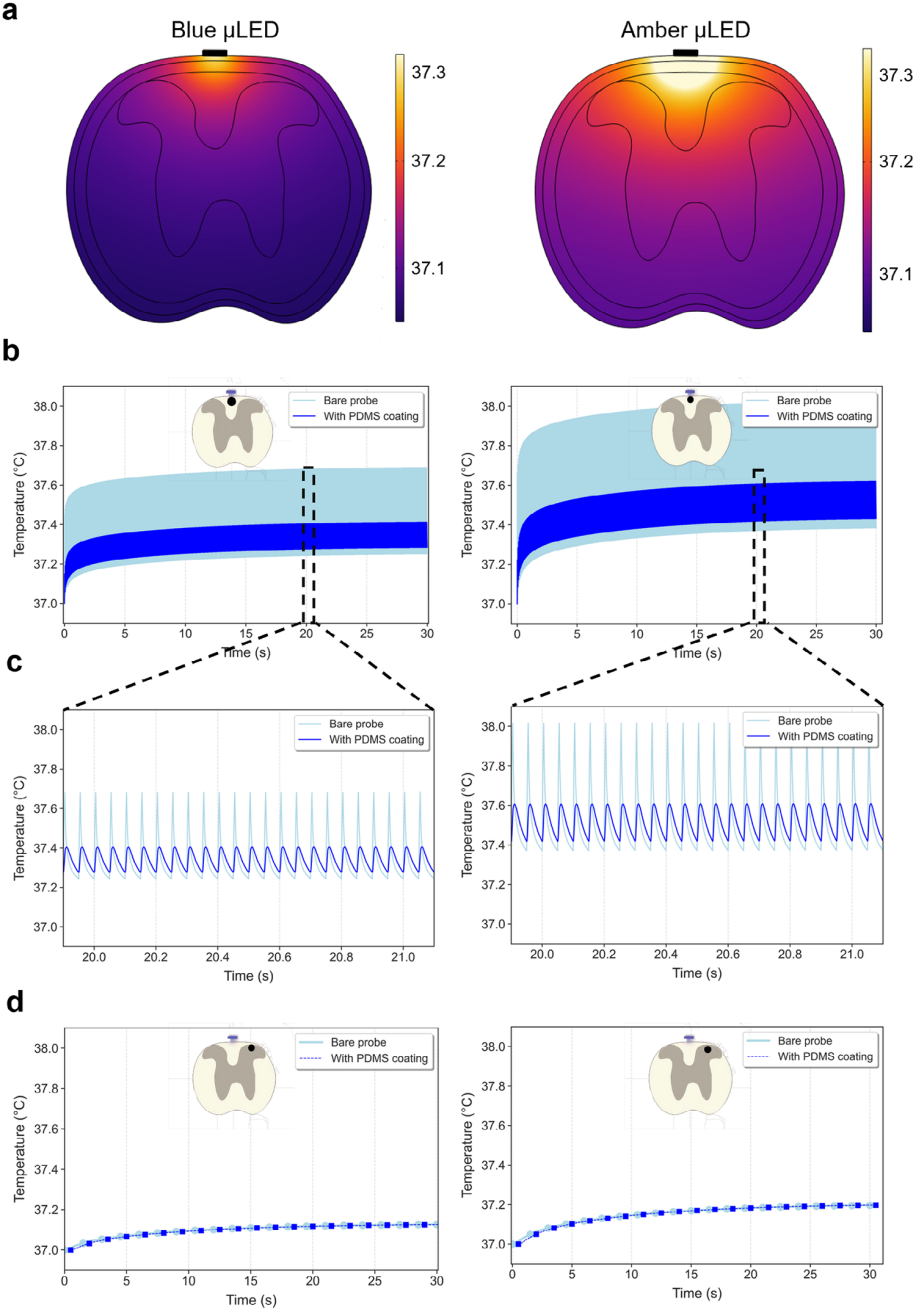
Thermal characterization of the light‐tissue interaction in mice. (a) Simulated steady‐state temperature distribution for blue (left) and amber (right) µLEDs after 30 s of continuous operation (electrical input power of 20 mW). (b,c) Temperature–time profiles at the spinal cord surface for blue (left) and amber (right) µLEDs with and without PDMS encapsulation over the initial 30 s (b) and from t = 20 s to t = 21 s (c), illustrating the thermal buffering effect of PDMS encapsulation. (d) Simulated temperature rise at the dorsal edge of the grey matter for blue (left) and amber (right) µLEDs with and without PDMS encapsulation.

To assess thermal safety, tissue heating was simulated during continuous optical stimulation. Figure 4a shows the temperature profile after stabilization for a µLED driven at an electrical input power of 20 mW for blue versus amber light. While blue light causes more heating at a point compared to amber light, the higher temperature rise with amber light is primarily attributed to the lower optical efficiency of the amber µLED (TCE10590) compared to the blue µLED (TR2227) used in this study. The computed thermal profile exhibits sinusoidal fluctuations driven by an on–off duty cycling of the µLED at the spinal cord surface with temperature rising during the initial ∼5 s and gradually stabilizing thereafter (Figure 4b). To assess the effect of PDMS coating on the thermal performance of the µLED probes, simulations were conducted for two configurations: one in which the µLED was encapsulated solely in a parylene‐C layer, and another with a dual‐layer structure comprising parylene‐C and PDMS. Notably, the PDMS layer acted as a thermal buffer, reducing peak temperatures and fluctuations by providing gradual heat dissipation (Figure 4b,c). The non‐encapsulated blue µLED exhibited a peak increase of > 0.7°C above baseline after stabilization, whereas the PDMS encapsulation caused the temperature rise to remain within 0.4°C (1°C and 0.6°C for the amber µLED, respectively). Furthermore, we specifically simulated the thermal heating at the dorsal edge of the grey matter where the neuronal cell bodies reside. The resulting temperature increases were approximately 0.16°C for the blue µLED and 0.2°C for the amber µLED and remained similar with or without PDMS encapsulation, which was attributed to the relatively large distance from the heat source (Figure 4d). This finding highlights the thermoregulatory properties of PDMS and its role in enhancing thermal safety for chronic implantation as well as providing mechanical compliance with the tissue. We repeated the thermal simulations using a rat spinal cord model, and the results were consistent with those obtained in mice, particularly with respect to the effects of PDMS encapsulation (Extended Data Figure S7).

### 2.6 Month‐Long Biological Performance and Stability of the Optoelectronic Device

To evaluate encapsulation durability, we conducted in vitro accelerated aging tests by submerging devices in stirred 1× PBS at 70°C (Extended Data Figure S8a). Devices retained full functionality for up to 16 days, after which intermittent declines in performance were observed. Post‐aging assessments confirmed the continued functionality of wireless charging and control circuitry. Also, the encapsulated batteries exhibited no signs of functional decline after the test. However, reduced optical output and inconsistent µLED activation were attributed to localized encapsulation failure at µLED soldering interfaces, despite the absence of macroscopic structural damage.

**FIGURE 5:**
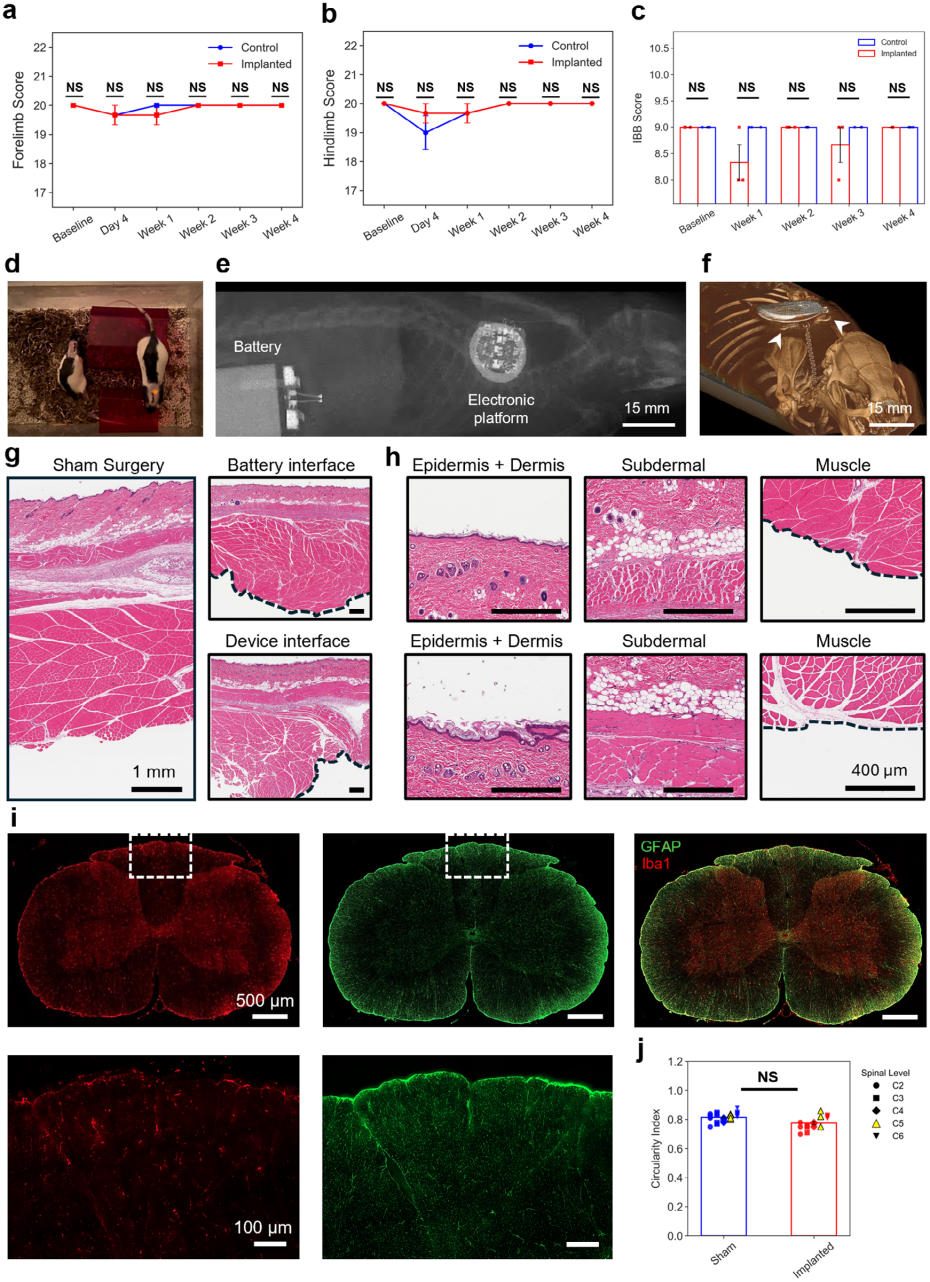
In vivo assessment of month‐long optoelectronic device implantation in rats. (a–c) Weekly behavioral assessments in rats over four weeks post‐implantation. Open‐field forelimb score (a), hindlimb score (b), and IBB forelimb function score (c) (*n* = 6 rats, Mann‐Whitney *U*‐test: *p* > 0.05, mean ± s.e.m.). (d) Photograph of two rats in the same cage, captured during a scheduled stimulation period when only one animal was receiving optical stimulation, while the other awaited its later session. (e) CT image showing implanted device location at 4 weeks post‐implantation. (f) Three‐dimensional CT reconstruction of the implanted optical probe placed at C5, 4 weeks post‐implantation. (g,h) H&E staining images of skin and muscle tissues at the implantation site in rats following sham surgery or device implantation (g), with corresponding zoomed view (h). (i) Immunohistochemical staining of the spinal cord at C5 after device implantation for Iba1 (red) and GFAP (green). (j) Quantitative assessment of the spinal cord circularity index in sham‐operated and device‐implanted rats (*n* = 9, two‐sided, unpaired Student's *t*‐test: p = 0.026 for C2, p = 0.029 for C3, p = 0.025 for C4, p = 0.835 for C5 and p = 0.113 for C6, mean ± s.e.m.).

To assess the month‐long biocompatibility and structural integrity of the implantable optoelectronic system, we performed chronic implantation studies in adult rats. Specifically, µLED probes were positioned at the cervical spinal cord, a region embedded deep within musculature and subject to greater dynamic mechanical strain, including extension and compression, compared to thoracic or lumbar segments [10] to rigorously evaluate their month‐long in vivo reliability. The µLED probes were designed to conform to the rat spinal cord anatomy [32] (length: 30 mm; width: 1.8 mm; height: 80 µm). Considering the available space between the dorsal skin and underlying musculature in rats, a 150 mAh battery (24 × 20 × 3.8 mm^3^) was selected for sustained operation. Following minimal dissection of the connective tissue between the vertebral laminae (inter and supraspinous ligaments), µLED probes containing two amber µLEDs (590 nm) at the tip and one indicator µLED at the base to verify successful device operation were inserted into the epidural space. As described in our previously published surgical protocol [33], the device was anchored to paraspinal muscle using sutures passed through two dedicated holes present in the periphery of the device platform, thereby minimizing micro displacements and reducing the risk of seroma formation (Figure 1h and Extended Data Figure S8b). To assess the impact of month‐long device implantation on animal behavior, we conducted weekly behavioral tests over 4‐week post‐implantation. No significant differences were observed between animals with and without implants across multiple behavioral paradigms, including open field locomotion assessment [34] (locomotor speed and gait pattern) (Figure 5a,b), and fine forelimb digit use during cereal handling using the Irvine, Beatties, and Bresnahan (IBB) [35] (Figure 5c). Figure 5d shows two rats housed together, with only one receiving optical stimulation at a scheduled time while the other remains unstimulated, demonstrating independent device control throughout the 4‐week study. 4 weeks post‐implantation, the animals were euthanized and perfused. Computed tomography (CT) imaging confirmed that the device components remained in their original implanted locations without detectable displacement (Figure 5e). Similarly, the µLED probe remained at the original location where it was implanted (Figure 5f). Following skin incision and tissue harvesting at the implantation site, gross examination did not reveal visible signs of damage to the surrounding skin, muscle, or electronics as well as the interconnection and the battery. Upon explantation, a thin encapsulating connective tissue layer was observed surrounding the device (Extended Data Figure S8c,d). Following device removal and cleaning, we verified the device full functionality after 4 weeks of implantation and extraction. In addition, hematoxylin and eosin (H&E) staining of the harvested tissue showed no overt pathological changes in these regions (Figure 5g,h). Notably, no adverse thermal effects or electromagnetic interference (EMI) with surrounding biological tissue were observed at the site of the receiver charging coil. To assess the probe‐spinal cord interface, immunohistochemical staining for ionized calcium‐binding adaptor molecule 1 (Iba1) and glial fibrillary acidic protein (GFAP) was performed on spinal cord segments C2–C6, revealing minimal inflammation at the interface (Figure 5i and Extended Data Figure S9a,b). Notably, the low Iba1 expression and branched morphology of the microglial cells suggest minimal microglial activation. Moreover, quantitative analysis of the spinal cord circularity did not reveal deviation from the geometry of the control animals, suggesting undetectable physical indentation caused by month‐long probe placement over the spinal cord (Figure 5j).

### 2.7 Evoking Optogenetic Activity in Freely Behaving Mice

Next, we investigated the utility of the devices by targeting the T10–T11 spinal segments, with the aim of inhibiting normal hindlimb locomotion in transgenic mice expressing channelrhodopsin‐2 (ChR2) under control of the VGAT (Slc32a1) promoter. This strain enables optogenetic activation of both GABAergic and glycinergic inhibitory interneurons in the spinal cord upon blue‐light stimulation [36]. The model broadly recruits inhibitory neurons known to exert rostro‐caudal suppression of hindlimb motor output when activated in the lower thoracic spinal cord [37]. We customized the µLED probe dimension (length: 19 mm; width: 1.2 mm; height: 80 µm) to meet the anatomical requirements for reaching the lower thoraco‐lumbar spinal segments in mice [38]. In parallel, a compact 12 mAh battery (9.5 × 9 × 3 mm^3^) was selected to provide the corresponding power demands (Extended Data Figure S2d). We demonstrated the capability of the device for its versatile use and its programmable nature at various times, periods, frequencies and durations. The optoelectronic devices were programmed to deliver 10 min of 50 Hz stimulation and 8 min of 20 Hz stimulation (470 nm, 5 ms pulses, 5 s on and 5 s off, 20 mW) during the morning and afternoon sessions, respectively, on day 4 post‐implantation (Supplementary Videos S7 and S8). Optical stimulation parameters were chosen based on previously reported in vivo results by Caggiano et al. [37]., *enabling* direct validation of the device performance under biologically established conditions (Supplementary Videos S6). To maximize power efficiency, the device was programmed to enter low‐power sleep mode during short periods of optical stimulation inactivity and to be awakened by scheduled alarms into low‐power run mode (Extended Data Figure S4b). The device was programmed to enter a low‐power standby mode with data retention after completion of an optical stimulation session, thereby reducing power consumption during extended periods (Figure 2l). Like the procedure described earlier for device implantation in rats, we inserted a µLED probe containing a single blue (470 nm) µLED at the tip through the L1‐L2 epidural space and advanced it smoothly to the targeted segment (T10). We assessed the impact of the two stimulation frequencies (50 and 20 Hz) on hindlimb motor function using the skilled horizontal ladder task (Figure 6a). Traversal time was significantly different between baseline and both 20 Hz and 50 Hz stimulation, whereas no significant difference was detected between the two stimulation frequencies, despite the 50 Hz group exhibiting a higher mean than the 20 Hz group (Figure 6b). Foot placement errors (missed steps) were also quantified (Supplementary Note 2), and a higher incidence of foot placement errors was observed during 50 Hz stimulation compared to 20 Hz (Figure 6c). Notably, 50 Hz stimulation resulted in major footfalls, whereas errors during 20 Hz stimulation were primarily associated with brief missteps, such as corrective paw adjustments or rapid, non‐weight‐bearing contacts. We also conducted repeated CatWalk tests to evaluate post‐implantation locomotor performance, confirming that the device size, weight, and implantation had no adverse effect on free movement. Gait parameters, including average speed and cadence during single runs, were assessed across 3 weeks (Figure 6d,e). Although cadence did not differ significantly over the 3‐week assay, a transient reduction in average speed was observed during the first week. This decrease was attributed to animals intermittent stopping during trials, likely reflecting postoperative stress. By weeks 2 and 3, average speed recovered to baseline levels. To further confirm gait stability, we analyzed the Step Regularity Index (SRI) (Figure 6f), which showed no evidence of abnormal stepping patterns at any point, including the first week (Supplementary Note 3). CT images acquired 3 weeks post‐implantation (Figure 6g) confirmed that both the device platform and the battery remained in their original positions throughout the study period. Reconstructed CT images (Figure 6h) further verified that the µLED probe remained positioned over the spinal cord at the same anatomical level (T10) of implantation. To assess potential pathological changes within the reduced subcutaneous space of mice compared to rats, we performed H&E staining after device explantation (Figure 6i,j). Consistent with the observations in rats, no significant damage was evident at the device–tissue interface. Moreover, we examined whether chronic probe placement induced changes in spinal cord morphology or elicited an immune response in mice. The low Iba1 and GFAP expression, together with the normal cellular morphology, indicated minimal microglial activation (Figure 6k,l). Furthermore, area quantification of Iba1 and GFAP positive staining showed no significant differences between probe‐implanted and sham‐operated spinal cords (Figure 6m). Quantitative analysis of spinal cord circularity (along L1 to T10) revealed no deviation from normal geometry (Figure 6n).

FIGURE 6Targeted optical stimulation of the spinal cord and inhibition of hindlimb locomotion in mice. (a) Chronophotography sequence of skilled horizontal ladder walking of a mouse during optical stimulation of the spinal cord, with blue shading indicating periods of stimulation. (b) Traversal time for ladder crossing under baseline, 20 Hz, and 50 Hz stimulation conditions (*n* = 4, Mixed‐effects model: baseline vs 20 Hz, p = 0.023; baseline vs 50 Hz, p = 3.5 × 10^−^^5^; 20 Hz vs 50 Hz, p = 0.062; mean ± s.e.m.). (c) Percentage of missed steps under baseline, 20 Hz, and 50 Hz stimulation conditions (*n* = 4, Mixed‐effects model: 20 Hz LED_on vs 20 Hz LED_off, p = 1.5 × 10^−^^4^; 50 Hz LED_on vs 50 Hz LED_off, p = 6.3 × 10^−^^8^; 20 Hz LED_on vs 50 Hz LED_on, p = 0.015, mean ± s.e.m.). (d) CatWalk gait analysis over 3 weeks post‐implantation showing average speed and cadence (*n* = 4, two‐sided paired Student's *t*‐test: p = 7.3 × 10^−^^3^ for speed at week 1, all comparisons were made against the baseline, mean ± s.e.m.). (e) Photograph of a mouse performing the CatWalk gait assay with corresponding step pattern analysis overlaid on the image. (f) Step Regularity Index (SRI) (*n* = 4, two‐sided paired Student's *t*‐test: p = 0.2901 for Week 1, p = 0.5115 for Week 2, p = 0.5774 for Week 3, mean ± s.e.m.). (g) CT image demonstrating in situ position of the device at 3 weeks post‐implantation. (h) Three‐dimensional CT reconstruction of the implanted optical probe at 3 weeks post‐implantation. The µLED position is indicated by a white arrow. (i,j) H&E staining of skin and muscle tissues at the implantation site in mice after sham surgery or device implantation (i), with corresponding zoomed view (j). Black arrows indicate connective tissue layers encapsulating the implant. (k,l) Immunohistochemical staining of the spinal cord at T10 following device implantation (k) and sham surgery (l), with Iba1 (red) and GFAP (green). (m) Quantification of Iba1 and GFAP immunostaining expressed as percentage of image area at T10 and T11 (*n* = 9, two‐sided unpaired Welch's *t*‐test; Iba1: T10, p = 0.672; T11, p = 0.607; GFAP: T10, p = 0.950; T11, p = 0.460, mean ± s.e.m.). (n) Quantitative assessment of the spinal cord circularity index in sham‐operated and device‐implanted mice (*n* = 9, two‐sided unpaired Student's *t*‐test: p = 0.493 for T10, p = 0.066 for T11, p = 0.516 for T12, p = 0.799 for T13 and p = 0.496 for L1, mean ± s.e.m.).

**Figure:**
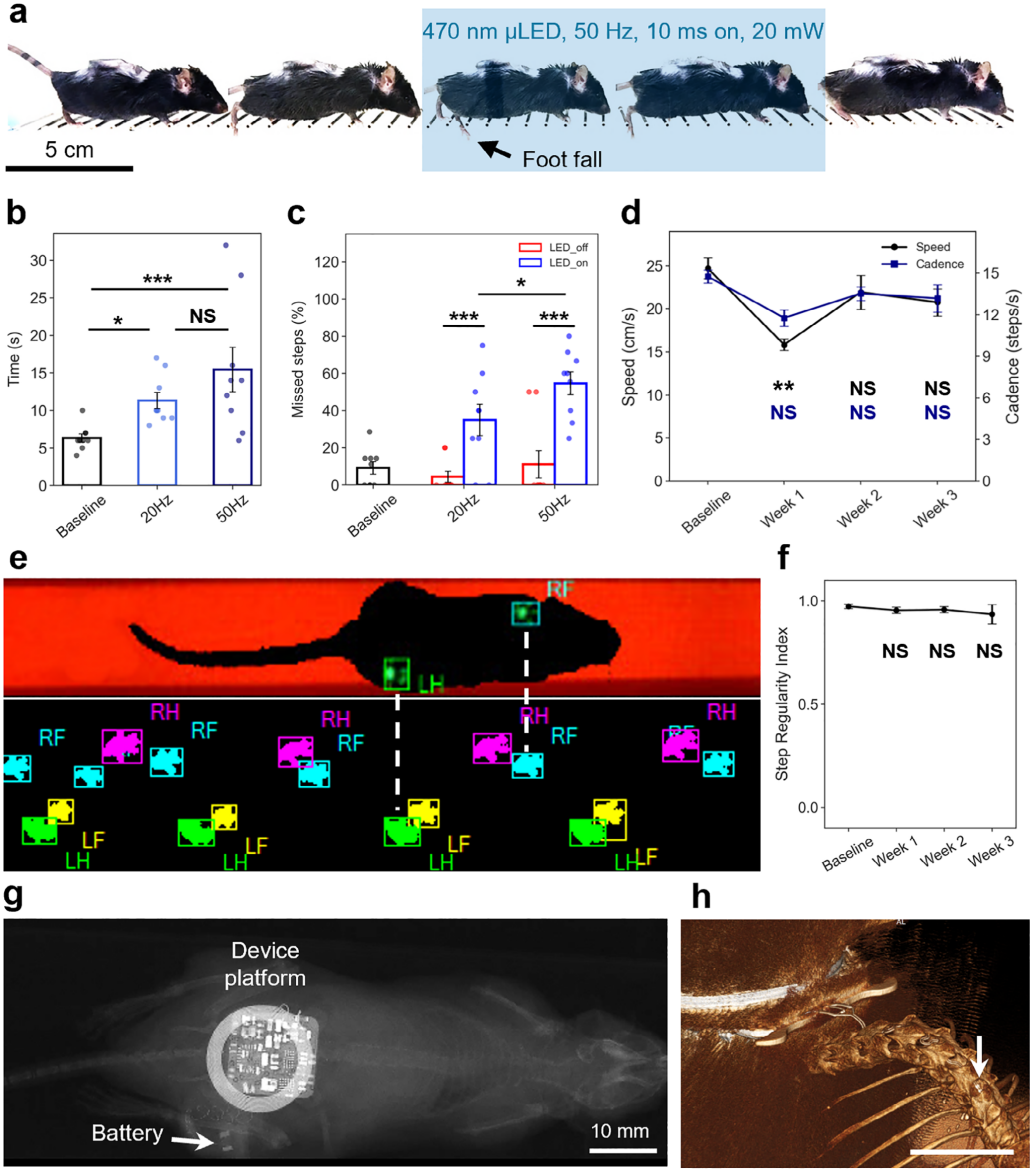

**Figure:**
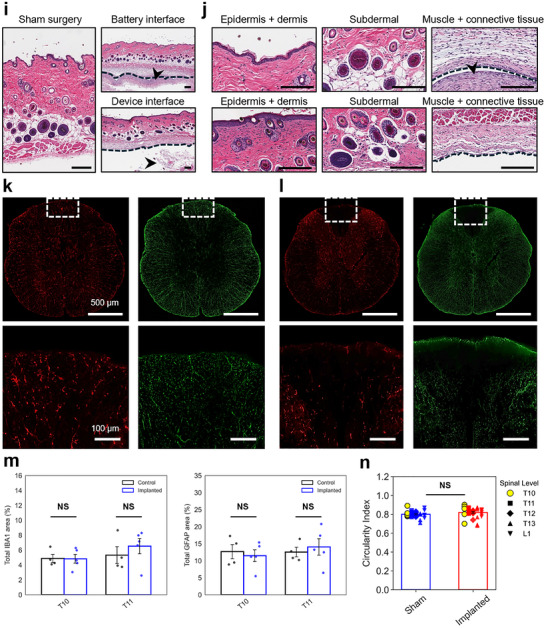

## 3 Discussion

We have reported a fully implantable optoelectronic system to deliver light to the spinal cord in freely moving rats and mice over extended periods. The system was engineered with modular architecture to reduce fabrication complexity and minimize the need to redesign across diverse experimental paradigms. The ultra‐low‐power design, featuring a wireless charging unit, an integrated low‐power calendar and optimized operating firmware, enables optical stimulation utilizing a small rechargeable battery over prolonged periods. To ensure accessibility, the system was developed as a low‐cost tool intended for broad adoption by the neuroscience community, without the need for electronics expertise or access to cleanroom facilities. Notably, we aimed to introduce a tool fabricated through industry‐compatible protocols, enabling large‐scale production to be outsourced while requiring only limited in‐house efforts for final assembly and programming. Specifically, the wireless charging interface is compatible with commercially available chargers in the market.

In addition, the optoelectronic device enables experimental paradigms that were previously constrained by battery‐less configurations or tethered headstage systems. Such constraints had limited the ability to conduct behavioral assays under naturalistic, unrestricted conditions. The fully implantable platform enables a wide range of behavioral studies, including open‐field locomotion, gait analysis, horizontal ladder crossing, and cereal handling (IBB) tasks, that were previously challenging to implement. Moreover, the system is well‐suited for months‐long optogenetic studies in freely behaving animals. For example, investigations of regeneration following spinal cord injury, chronic neuromodulation for neuropathic pain and modulation of locomotor patterns in neurodegenerative disease models [26]. Apart from research applications, recent clinical trials have commenced to evaluate the therapeutic potential of optogenetics in humans [39]. The spinal cord is recognized as a primary target for optogenetic interventions, owing to its anatomical accessibility and the potential to address unmet clinical needs in neuromodulation and neural circuit restoration [26]. With features such as full implantability, wireless charging, and programmable stimulation, these devices could fulfill critical design requirements for clinical use and can be adapted for human spinal cord applications. In the current implementation, the µLED drive current is set by a fixed hardware resistor and is user‐configurable prior to insulation and implantation. Future iterations of the system could incorporate wireless communication capabilities, enabling real‐time control over optical stimulation parameters and expanding its functionality for closed‐loop or adaptive stimulation paradigms.

## 4 Methods

### 4.1 Micro‐LED Probe Assembly and Encapsulation

The flexible µLED probes were designed as a multilayer structure comprising a single‐layer copper trace patterned on a PI substrate, with a second PI layer functioning as the soldermask. Designated copper pads were used for precise alignment and soldering of µLEDs (TCE10, III‐V compounds, for emission at 590 nm; TR2227, CREE, for emission at 470 nm). Encapsulation of the fully assembled µLED probes began with a 5 µm conformal coating of parylene‐C, deposited via chemical vapor deposition (PDS 2010, Specialty Coating Systems). In parallel, standard glass slides were cleaned with isopropyl alcohol (IPA), followed by N_2_ blow‐drying. A base layer of PDMS (20 µm) was then formed by spin‐coating at 2500 r.p.m. for 35 s, followed by curing at 80°C for 20 min. The µLED probes were then positioned on the cured PDMS base, and a second PDMS layer (50 µm) was formed by spin‐coating at 1200 r.p.m. for 30 s. An extended curing process at 80°C for an additional 80 min completed the encapsulation process. The µLED probes were then cut to their final dimensions and gently peeled from the glass slide. These steps are summarized in Extended Data Figure S10a. Finally, electrical pads were exposed by mechanically scraping the parylene and PDMS layers with a razor blade, and the functionality was verified before integration with the electronic core. To ensure mechanical stability and electrical hermeticity at the connection between the flexible probe and the electronic core, an epoxy adhesive (2 Ton, Devcon) was applied.

### 4.2 Optoelectronic Device

#### 4.2.1 Electronic Core Platform

The electronic core was designed as a compact four‐layer PCB fabricated on an *FR4* substrate. The *layer stack‐up* included a dedicated internal ground plane to shield the signal tracks against high‐intensity electromagnetic interference (EMI) associated with Qi‐based wireless power transfer and to enhance signal integrity (Extended Data Figure S10b). The layout incorporated analog, digital, and power management circuitry, with a minimum trace‐to‐trace clearance of 50 µm to support high‐density routing within the constrained implantable form factor. The PCB was fabricated by a commercial vendor (PCBGOGO) using 0.5 oz. copper thickness standard. The final board dimensions were 14 × 13 mm^2^, with a total thickness of 400 µm including all conductive and dielectric layers. Surface‐mount components were assembled using a reflow soldering process. A 32‐bit ARM Cortex‐M0+ ultra‐low‐power microcontroller (STM32L031G6U6S, STMicroelectronics) served as the central control unit. A low‐power 32.768 kHz quartz crystal oscillator (ECS‐.327‐7‐12R, ECS Inc.) was integrated as the clock source to enable real‐time scheduling of µLED activation. Independent control of the µLEDs was achieved using two programmable LED drivers (PAM2804, Diodes Inc.), enabling stable and individually regulated light intensity for each set of µLEDs. Prior to implantation, the assembled devices were encapsulated using a dual‐layer coating: a 12 µm conformal layer of parylene‐C deposited via chemical vapor deposition (PDS 2010, Specialty Coating Systems) to provide a uniform moisture barrier, followed by a dip‐coated outer layer of PDMS (Sylgard 184, Dow Corning) for 400–600 µm (Supplementary Note S4).

#### 4.2.2 Wireless Charging Module

Wireless power transfer was implemented via a resonant inductive coupling circuit, consisting of a receiver coil paired with a dual‐capacitor network sized correctly per the *WPC v1.2* specification [40]. Resonant capacitances, *C1* and *C2*, were calculated using equations (1) and (2), respectively.

(1) $$C_{1}=\frac{1}{{\left(2\pi f_{s}\right)}^{2}L^{\prime }}$$

and

(2) $$C_{2}=\frac{1}{{\left(f_{d}\times 2\pi \right)}^{2}\times L-\frac{1}{C_{1}}}$$

where *L*′ is the measured coil inductance in the final setup, *L* represents the free‐space coil inductance, *f_d_* is 1 MHz ± 10% and *f_r_* is the *LC* resonant frequency (110 kHz). An integrated Qi‐compliant power receiver (BQ51013B, Texas Instruments) was used to manage power regulation and communication control with the power transmitter. The receiver antenna (WR202010, TDK Corp) was selected to match the dimensional constraints of the final assembled device. A ferrite shielding layer (IFL05K, TDK Corp) was patterned using a laser ablation system (ProtoLaser U4, LPKF) and integrated to reduce electromagnetic interference. The rectified wireless power was routed to a battery charger with powerpath management (BQ24253, Texas Instruments). The battery charger managed battery charging and regulated power distribution to the circuitry, which enabled source selection and provided protection against electrical shorts. The wireless charging was performed using a commercially available wireless charger (MagSlim Wireless Charger, Nillkin).

### 4.3 Adjustable Soft Interconnections Design and Preparation

The soft interconnections consisted of PI‐Cu‐PI‐Cu‐PI (12.5 µm – 18 µm – 25 µm – 18 µm – 12.5 µm) *stack up*. Interconnection lengths were customized based on animal size to accommodate anatomical variability across mice and rats. To prepare the soft interconnections for implantation, the fPCBs were cleaned with IPA, followed by N_2_ blow dry. A base layer of silicone elastomer (Ecoflex 00‐30, Smooth‐On) was pre‐cast in a Petri dish (∼150 µm) and cured at 100°C for 30 min, forming a uniform layer. The interconnections were positioned on the cured base layer and gently pressed using the tip of forceps to ensure uniform contact. A second layer of uncured silicone (Ecoflex 00‐30, Smooth‐On) was then poured (∼500 µm) to fully encapsulate the structures. The assembly was left undisturbed on a levelled surface for 10 min to let the silicone layer flatten, followed by curing at 100°C for 45 min. After curing, the encapsulated interconnections were cut to their final dimensions. Electrical connection pads at both ends of the interconnections were exposed and soldered to the device and the battery terminals. A layer of epoxy adhesive (2 Ton, Devcon) was first applied to the solder joints to provide moisture resistance and mechanical reinforcement. This was followed by an additional coating of silicone adhesive (Sil‐Poxy, Smooth‐On) to further stabilize the solder joints and improve long‐term durability.

### 4.4 Battery Encapsulation

Lithium‐polymer (Li‐Po) batteries were encapsulated in custom‐designed enclosures fabricated from a biomedical‐grade resin (BioMed Clear, Formlabs Inc.). Enclosure designs were created in Autodesk Fusion 360 and processed using the commercial software PreForm (Formlabs Inc.) for 3D printing. Final structures were printed using a stereolithography (SLA) 3D printer (Form 2, Formlabs Inc.). Upon completion of printing, the parts were carefully removed from the build platform and washed twice in IPA for 15 min to eliminate residual uncured resin. The printed components were then placed under a chemical fume hood to let air dry for 45 min. The functional pieces were post‐cured in a UV curing chamber (Formlabs Inc.) at 60°C for 60 min to complete polymerization and enhance mechanical strength. The cured enclosures were inspected for structural integrity and any defects. Each battery was then placed inside the enclosure and sealed using additional resin (BioMed Clear, Formlabs Inc.) to provide fluid‐tight encapsulation.

### 4.5 Thermal and Optical Modelling

Light–tissue interaction in the spinal cord was modelled in COMSOL Multiphysics (v6.1, COMSOL Inc.). A finite element analysis (FEA) was implemented to simulate optical field propagation and generate heat distribution within the spinal cord. Two‐dimensional transverse cross‐sectional geometries were generated in SolidWorks (Dassault Systèmes) from anatomical data extracted from rat and mouse spinal cord atlases [32, 38] and were imported into COMSOL for simulation. The light penetration was calculated under steady‐state conditions by solving the Helmholtz equation (equation (3)),

(3) $$\nabla \cdot \left(-c\nabla \phi \right)+\mu_{a}\phi =f$$

where *ϕ* represents the light fluence rate in the spinal cord, *c* is the diffusion coefficient, *µ_a_* is the absorption coefficient, and *f* is the source term. The isotropic diffusion coefficient is defined as shown in equation (4),

(4) $$c=1/3(\mu_{a}+\mu_{s}\prime )$$

which 𝜇_s_′ is the reduced scattering coefficient and can be calculated using the relation 𝜇_s_′= 𝜇_s_ (1 − *g*), where 𝜇_s_ is the scattering coefficient and *g* is the anisotropy factor [41]. Optical parameters of the model (absorption coefficient, scattering coefficient, and anisotropy factor) for different spinal cord tissue layers at two wavelengths, amber (590 nm) and blue (470 nm), were assigned based on values previously reported in the literature [42], as shown in Extended Data Figure S5b. A parametric sweep was performed across a range of µLEDs electrical input powers (10, 20, 40, 60, and 80 mW). To map electrical input power to optical output, reported optical efficiencies from the literature were applied for blue (36.1%) and amber (6.4%) µLEDs used in this study [11, 43, 44].

To quantify the temperature profile of the spinal cord tissue during optical stimulation, time‐dependent bioheat transfer equations were employed. The transient heat transport was modelled using the bioheat equation, as expressed in equation (5):

(5) $$\rho C_{p}\partial T/\partial t+\nabla \cdot \left(-k\nabla T\right)=\rho_{b}C_{b}\omega_{b}\left(T_{b}-T\right)+Q_{met}+Q_{the}+\phi \mu_{a}$$

where *T* is temperature, *t* is time; *k*, *ρ* and *C_p_* are the thermal conductivity, mass density and specific heat capacity of the spinal cord, and *ρ_b_* and *C_b_* are the mass density and specific heat capacity of the blood, respectively. The parameter *ω_b_* denotes the blood perfusion rate, and *T_b_* is the arterial blood temperature. *Q_met_* represents the heat source from metabolism in the spinal cord, *Q_the_* is the heat generated by the µLEDs during stimulation and was calculated from the electrical input power by accounting for the fraction of power converted to heat, defined as 1 − η_opt_, where η_opt_is the literature‐reported optical efficiency of blue (36.1%) and amber (6.4%) µLEDs used in this study [11, 43, 44]. ***ϕ*** is the optical fluence rate, and *µa* is the absorption coefficient [45]. The thermal power of µLEDs was modelled by defining a line heat source embedded in the PDMS layer positioned above the epidural fat. Thermal properties of the spinal cord tissue and bio‐heat input parameters used in the simulations were obtained from previously published reports [46] and are summarized in Extended Data Figure S5b.

### 4.6 Animal Models

All animal studies were conducted on adult female Long Evans rats (200–300 g; Charles River Laboratories, strain 006) and adult male VGAT‐ChR2‐YFP BAC transgenic mice (28–32 g; Jackson Laboratory, stock #014548). Animals were housed under a 12‐h reverse light‐dark cycle with ad libitum access to food and water. All animal procedures were approved by the University of British Columbia Animal Care Committee (approval number A21‐0299) and were conducted in accordance with the guidelines of the Canadian Council on Animal Care. Animals were monitored and cared for twice daily during the first week post‐surgery, and subsequently once daily until the experimental endpoint.

### 4.7 Surgical Procedure

We have previously described general surgical procedures in detail [33]. In both rats and mice, anesthesia was induced with 5% isoflurane and maintained at 2% in oxygen (flow rate: 1–1.5 L/min). Analgesic buprenorphine (Vetergesic Injectable, catalog no. 124918) was given prior to the surgery and twice daily for 3 days after surgery (0.03–0.05 mg/kg). In rats, the back of the neck was shaved, disinfected, and the skin was cut longitudinally, and the muscles dorsal to the spinal column were split along the midline. The interspinous ligament C6‐C7 was resected, and the µLED probe was advanced into the epidural space beneath the C6 lamina, positioning the µLEDs above the C5 spinal segment. The device platform was then sutured to the adjacent musculature using a nylon 6‐0 suture (Synergy Surgical, catalog no. N6021).

In mice, we identified vertebral level T13 by palpating the most caudal pair of ribs and their articulation with the spinal column. A midline skin incision was made with T13 as the central reference point. L1 and L2 were then identified by counting caudally from T13, and the connective tissue between these vertebrae was cleared to expose the underlying spinal cord. A subcutaneous pocket was created caudal to the incision using a hemostat to bluntly separate the skin from the underlying muscle. The device was placed into the pocket, and the µLED probe was inserted epidurally over the spinal cord by guiding it beneath the lamina at the L1‐L2 intervertebral junction. The device platform was then anchored by suturing (Synergy Surgical, catalog no. N6021) to the adjacent musculature. After surgery, animals were transferred to a warm recovery incubator.

### 4.8 Computer Topography Imaging

All imaging was performed post‐euthanasia. Images were acquired using a preclinical micro‐CT system (VIVACT40, Scanco Medical) with a 38.9 mm field of view, operated at 70kVp and 114 µA over a 360° rotation. Animals were positioned in an imaging tube with the body elongated and the dorsal side facing upward. Projection Data were reconstructed at a voxel size of 38 µm using a filtered back‐projection software suite (Scanco Medical). Following reconstruction, the DICOM datasets were processed using a custom‐written MATLAB script to enhance image contrast and remove background noise, then imported into RadiAnt DICOM Viewer (Medixant) to generate 3D models of the spinal cord and surrounding structures for qualitative visualization and further morphometric analysis. System calibration was performed twice: first with a phantom limb at the start of the imaging session, and subsequently before each scan using an empty chamber to ensure X‐ray measurement accuracy.

### 4.9 Circularity Index

Calculating the circularity index of the spinal cord cross‐sections was manually performed using ImageJ software (v1.53, National Institutes of Health). Histological cross‐sectional images of the spinal cord were converted to 8‐bit grayscale, and binary masks were generated by setting a proper threshold. The spinal cord boundary was delineated, and the circularity index was automatically calculated by ImageJ according to equation (6):

(6) $$CI=\frac{4A}{\left(\pi \times D^{2}\right)}$$

where *A* denotes cross‐sectional area and *D* represents the length of the major axis of the spinal cord.

### 4.10 Data Analysis

All data analysis and visualization were performed using custom‐written Python scripts (v3.12, Python Software Foundation) incorporating the NumPy, Pandas, SciPy, and Matplotlib libraries. Results are presented as mean ± s.e.m. Behavioral measurements were repeated across multiple trials (3–8 repetitions per experiment, depending on the specific assay). Data from failed or malfunctioning devices were excluded from the analysis. Normality of data distributions was assessed prior to analysis using the Shapiro–Wilk test and additional diagnostic checks. Statistical analyses were conducted using paired or unpaired Student's *t*‐test, Mann–Whitney *U*‐test, and linear mixed‐effects models, applied as appropriate. For multi‐group comparisons, post‐hoc pairwise contrasts with Holm/Holm–Šidák correction were applied where applicable. For all analyses, a significance level of *p* < 0.05 was set and “NS” indicates p > 0.05. Plots were generated with error bars representing s.e.m., and individual data points were overlaid for transparency.

### 4.11 Animal Behavioral Study

#### 4.11.1 Horizontal Ladder

The horizontal ladder task was performed following established protocols previously described in the literature [47]. Briefly, the mice were placed at one end of a horizontal ladder with evenly spaced rungs and video‐recorded while allowed to traverse to the opposite end, motivated by their home cage. Hindlimb placements were individually scored on a 0–6 scale based on paw placement accuracy (Supplementary Note 2). For missed‐steps percentage calculations, scores of 0 to 4 were classified as misses, while scores of 5 and 6 were considered hits.

#### 4.11.2 The Irvine, Beatties and Bresnahan Forelimb Scale

Animals were placed individually in a transparent cylinder with cereal scattered on the floor to encourage forelimb use. Mirrors were arranged around the apparatus to enable unobstructed observation from all angles. Digit usage during spontaneous cereal handling was recorded and subsequently scored using the IBB forelimb assessment method, as previously described [48].

#### 4.11.3 Gait Analysis

Gait analysis was conducted using the CatWalk XT automated gait system (Noldus Information Technology). Animals were acclimated to the system during the week preceding data collection, with three 10‐min sessions per day. Baseline gait recordings were obtained 2–3 days prior to the device implantation surgery. Following implantation of the optoelectronic device, animals were tested once per week for 21 days. A trial run was considered compliant if the duration was between 0.5 and 10 s and the maximum variation in speed did not exceed 60%. Standard mouse calibration [49] was used, including predefined settings for camera gain, green light intensity, analysis window size, and thresholds for run duration and speed variation. Non‐compliant runs were automatically flagged by the software and excluded from analysis.

### 4.12 Animal Perfusion and Immunohistology

#### 4.12.1 Perfusions

At the experimental endpoint, animals were deeply anesthetized with 5% isoflurane in oxygen and euthanized with an intraperitoneal injection of an overdose of chloral hydrate, followed by diaphragm transection when going into respiratory arrest. Transcardial perfusion was performed with 1× PBS, followed by 4% paraformaldehyde (PFA) in 1× PBS for 10 min (20 mL for mice and 200 mL for rats). Tissue was post‐fixed in 4% PFA for 24 h, washed in 1× PBS for 24 h, and cryoprotected in 30% sucrose for 72 h prior to sectioning.

#### 4.12.2 Immunohistochemistry

Following perfusion, tissue from the relevant spinal cord levels was embedded in optimal cutting temperature (O.C.T.) compound and snap frozen. Transverse sections were cut at 20 µm thickness using a microtome (HM525 NX, Thermo Scientific), mounted directly on glass slides, and air‐dried at room temperature. Sections were blocked for 30 min in normal donkey serum (Jackson ImmunoResearch, catalog no. 017‐000‐121) and incubated overnight at room temperature with the following primary antibodies: rabbit anti‐GFAP (1:1000, Agilent Technologies, catalog no. Z0334) and chicken anti‐Iba1 (1:500, Aves Labs, catalog no. IBA1‐0100). After three 10‐min washes in 1× PBS, appropriate secondary antibodies: donkey anti‐rabbit Alexa Fluor 488 (Invitrogen, catalog no. A‐21206), donkey anti‐chicken Alexa Fluor 594 (Invitrogen, catalog no. A78951), donkey anti‐rabbit Alexa Fluor 594 (catalog no. 711‐585‐152, Jackson ImmunoResearch), donkey anti‐chicken Alexa Fluor 647 (Invitrogen, catalog no. A78952) were applied for 2 h at room temperature. Slides were washed again and subsequently cover‐slipped using Fluoromount‐G with DAPI (catalog no. 00495952, Invitrogen). Confocal images were acquired using a confocal microscope (Zeiss Axio‐Observer M1 equipped with Yokogawa spinning disc) and processed using ImageJ.

#### 4.12.3 Hematoxylin and Eosin Staining

Tissues surrounding the device platform and battery were harvested following perfusion, as previously described. The overlying skin and muscle were excised and post‐fixed in 4% PFA overnight at 4°C, followed by a 24‐h wash in 1× PBS. Samples were then transferred to 70% ethanol for storage prior to paraffin embedding. Serial sections were cut at 6 µm thickness and mounted on slides for histological analysis. H&E staining was conducted using a standard protocol. Brightfield images were acquired at 20× magnification using a slide scanner (Aperio CS2, Leica Biosystems). Tissue samples obtained from areas without direct contact with any device components served as controls for comparative analysis.

### 4.13 Accelerated Ageing Assay

Accelerated aging tests were conducted to evaluate the long‐term stability and functional lifespan of the optoelectronic device under physiologically relevant conditions. Devices were submerged in 1× PBS and placed in a sealed Petri dish on a hot plate maintained at 70°C. To mimic continuous fluid dynamics and to prevent concentration gradients and thermal layering, the solution was agitated at 300 RPM using a magnetic stir bar. Devices remained continuously immersed throughout the testing period and were visually inspected and functionally evaluated on a daily basis.

### 4.14 Mechanical Testing

Mechanical characterization of soft interconnections was performed using a uniaxial tensile testing machine (Dual Column Tabletop 5969, Instron) equipped with a 10N load cell to assess both the cyclic deformation behavior and ultimate tensile properties of interconnections. For the tensile failure tests, samples were extended at a constant displacement rate of 1.5 mm/s until mechanical failure occurred. Data was recorded at 100 ms intervals with a load resolution of 1N. For the cyclic tensile tests, samples were mounted with an initial length (L_1_) of 20 mm and subjected to a 10 s preloading hold. The samples were then cyclically extended with 25% elongation at a rate of 10 mm/s (1 Hz) for 20 000 loading–unloading cycles under displacement‐controlled mode. All tests were conducted at room temperature.

## Funding

This work was supported by the Government of Canada New Frontiers in Research Fund–Transformation (NFRFT‐2020‐00238), the Canadian Institutes of Health Research (grant no. 191895), the Michael Smith Health Research BC Scholar Award (to D.S), and the University of British Columbia Four Year Doctoral Fellowship (to S.S).

## Conflicts of Interest

The authors declare no conflicts of interest.

## Supporting information




**Supporting File 1**: advs75419‐sup‐0001‐SuppMat.docx.

**Supporting File 2**: advs75419‐sup‐0002‐VideoS1‐S9.zip.

## Data Availability

The data that support the findings of this study are available from the corresponding author upon reasonable request.
