# Supplementary material for: Wireless, Adaptable and Fully Implantable Battery‐powered Devices for Optical Stimulation of the Spinal Cord in Small Rodents
Source: Adv Sci (Weinh). 2026 Apr 22;13(40):e75419. doi: 10.1002/advs.75419 (PMC13335532; doi:10.1002/advs.75419)
Supplement: Supplementary file 1 — Supporting File 1: advs75419‐sup‐0001‐SuppMat.docx. [file ADVS-13-e75419-s001.docx]

**Extended Data Figures**

**
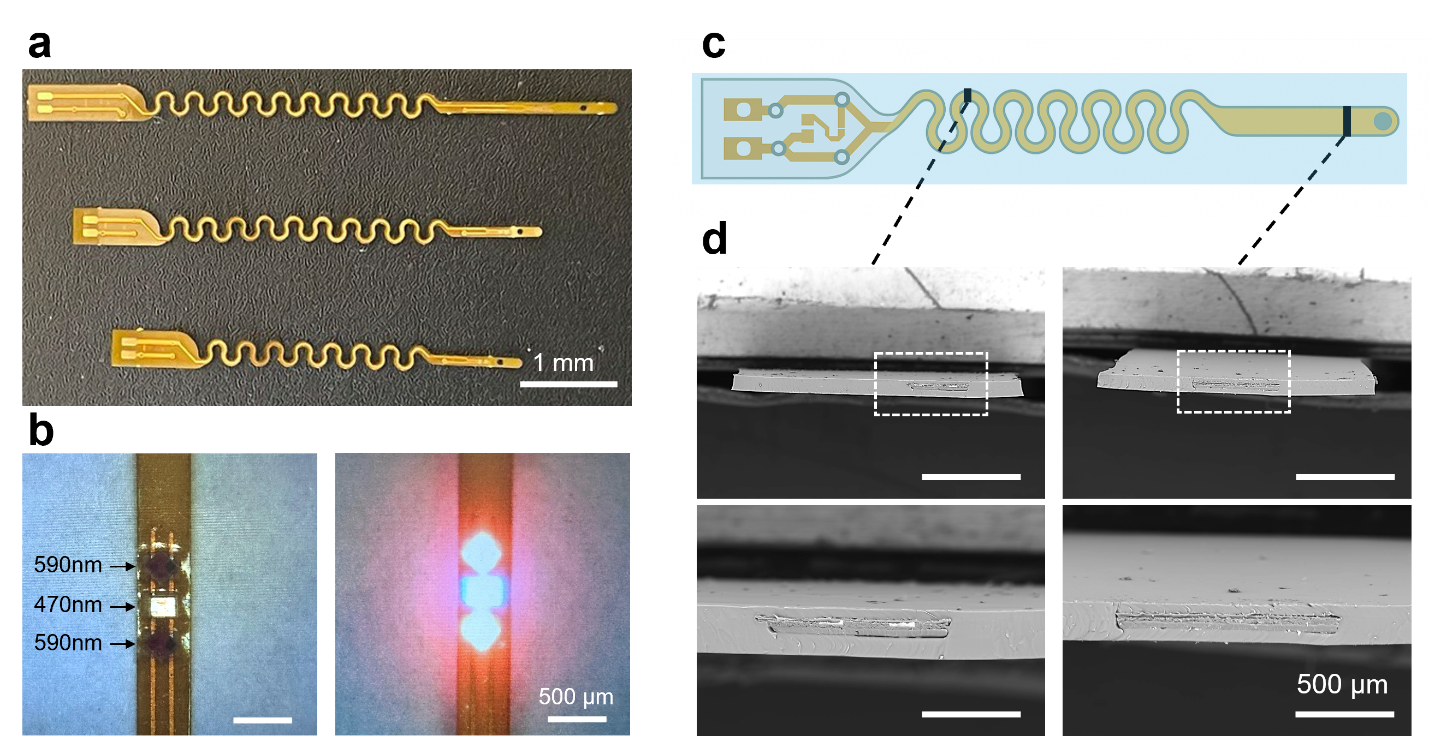
**

**Extended Data Fig. 1 | Soft, flexible micro-LED probes with structural variations and customizable wavelength emission. a,b** Optical image of µLED probes fabricated in different lengths, widths, and serpentine geometries (**a**) and zoomed-view images of a triple-µLED configuration (590 nm/470 nm/590 nm) (**b**), demonstrating wavelength-specific emission for multi-opsin modulation (bottom). **c**, Schematic illustration of the encapsulated µLED probe layout. **d,** Cross-sectional SEM images at the serpentine region (left) and probe tip (right), confirming uniform encapsulation of the µLED probes.

**
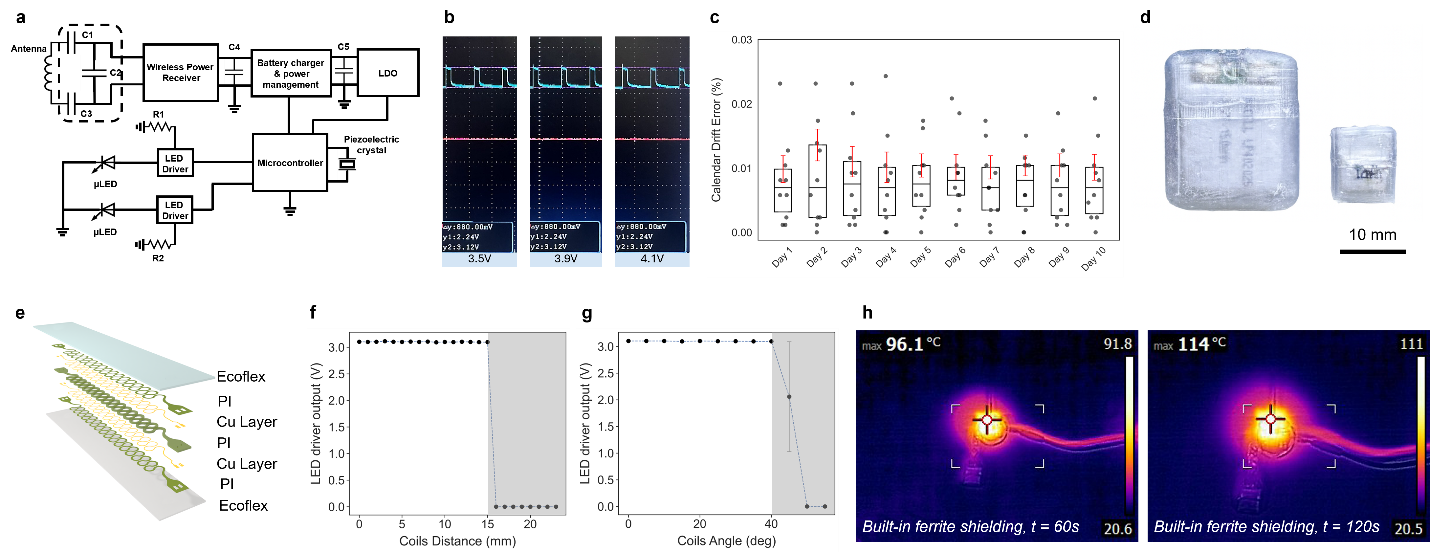
**

**Extended Data Fig. 2 | Optoelectronic device design, performance stability, and wireless charging** **characterization. a,** Electrical schematic of the electronic core illustrating the control unit, power management, wireless charging unit and µLED driving circuitry. **b**, Representative oscilloscope traces showing LED driver output driving a single blue light (470 nm) µLED (programmed to deliver 20 mW) across different battery voltage levels (3.5–4.1 V). **c**, Calendar unit drift error measured over 10 days under physiological conditions (*n* = 10, mean ± s.e.m.). **d**, Two batteries of different capacities encapsulated in 3D-printed, medical-grade photopolymer cases. **e**, Exploded-view schematic of the flexible interconnection, with a double-layer fPCB encapsulated between two EcoFlex layers. **f**,**g**, LED driver output voltage during wireless charging of the battery as a function of lateral coil displacement (**f**) and angular offset (**g**), indicating tolerance up to 15 mm and ~45°, respectively (*n* = 3, mean ± s.e.m.). **h**, Infrared thermography of the device **during wireless charging** of the battery with the **unmodified receiver antenna**, showing a localized hotspot near the power‑management electronics peaking at >95°C within 60 s and >110°C within 120 s.

**
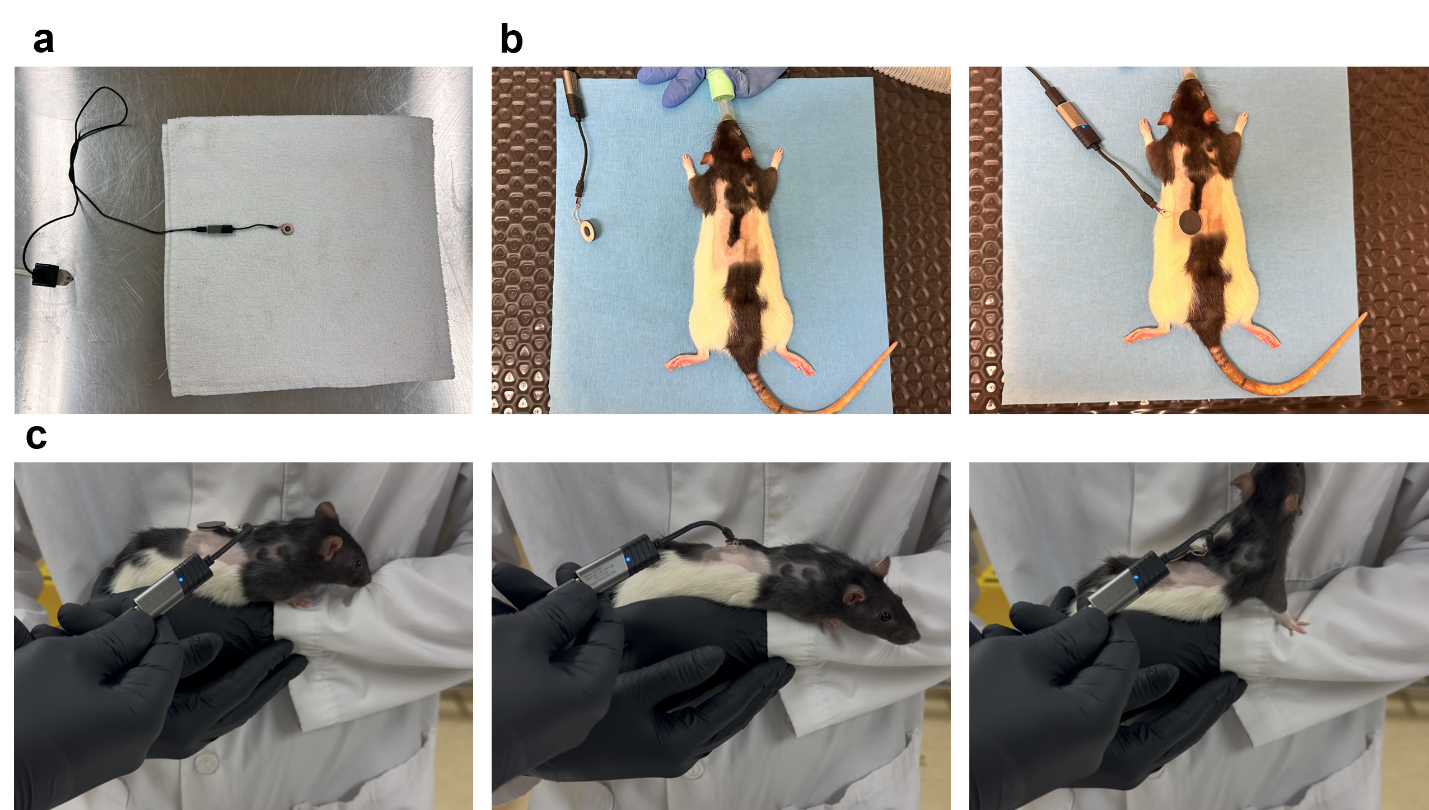
**

**Extended Data Fig. 3 | Wireless charging of implanted optoelectronic devices *in vivo*. a**, Experimental setup for the wireless charging of the devices using a commercially available charger, with a small magnet attached to the transmitter antenna and a warm blanket used for animal placement. **b**, Wireless charging of an implanted device in an anesthetized rat placed on a warm blanket, with transmitter–receiver coil alignment facilitated by the magnet attached to the transmitter. The indicator LED on the wireless charger confirms real-time power transmission (on during power delivery and off when transmission is absent). **c**, Wireless charging of implanted devices in conscious rats, demonstrating stable power transmission during free handling of the animal.

**
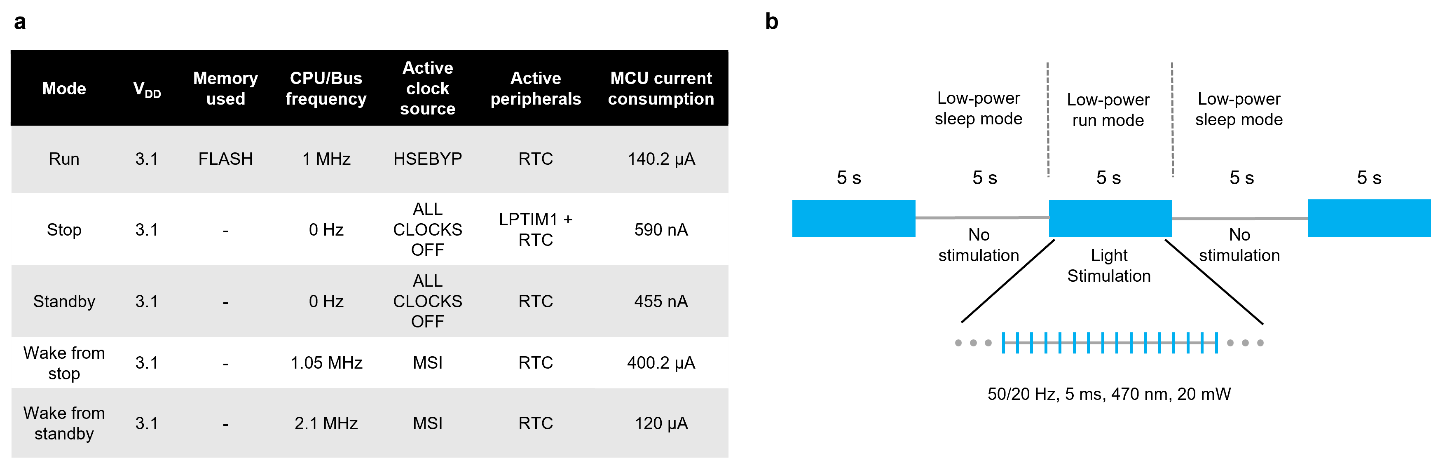
**

**Extended Data Fig. 4 | Various power modes of the optoelectronic device and programmable optical stimulation. a**, Power consumption profile of the MCU across different programmable modes, including active run, stop, standby, and wake-up states, with corresponding current levels. The values were derived from the STM32L031G6U6 datasheet (DS10668_Rev4) at room temperature (25 °C). **b**, Schematic representation of device power-mode transitions during optical stimulation. The device enters low-power sleep mode during inactive intervals and is awakened by scheduled alarms into low-power run mode for light delivery continuation.

**
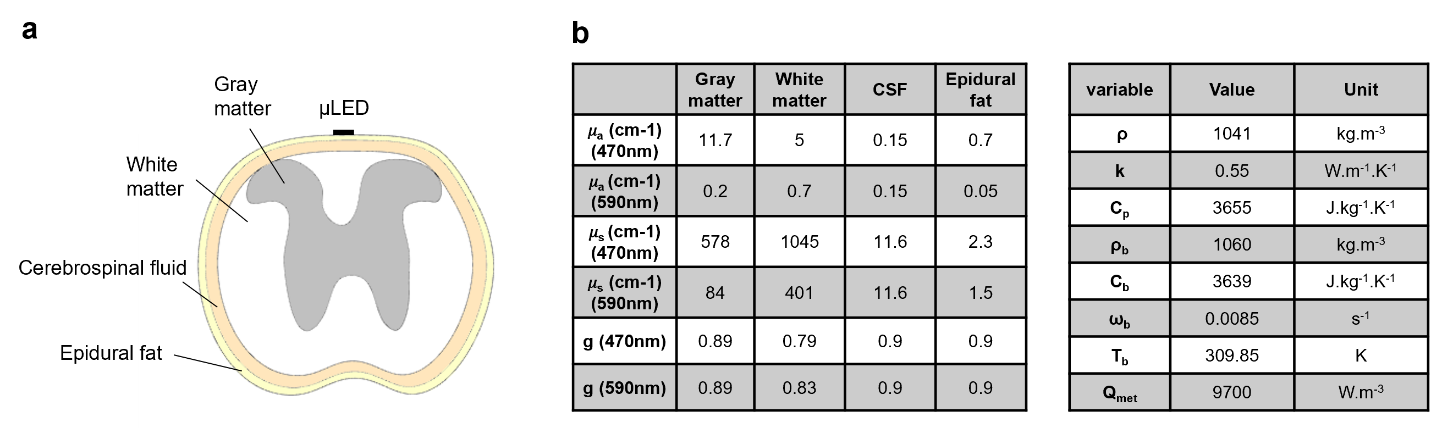
**

**Extended Data Fig. 5 | Computational model of spinal cord anatomy and assigned optical and thermal parameters. a**, Schematic cross-section of the spinal cord illustrating distinct anatomical layers incorporated into the computational model, including grey matter, white matter, cerebrospinal fluid (CSF), and epidural fat. **b**, Optical properties of each tissue layer at 470 nm (blue) and 590 nm (amber) wavelengths (left) and thermal properties of the spinal cord tissue with bio-heat model input parameters (right), derived from previously reported values.

**
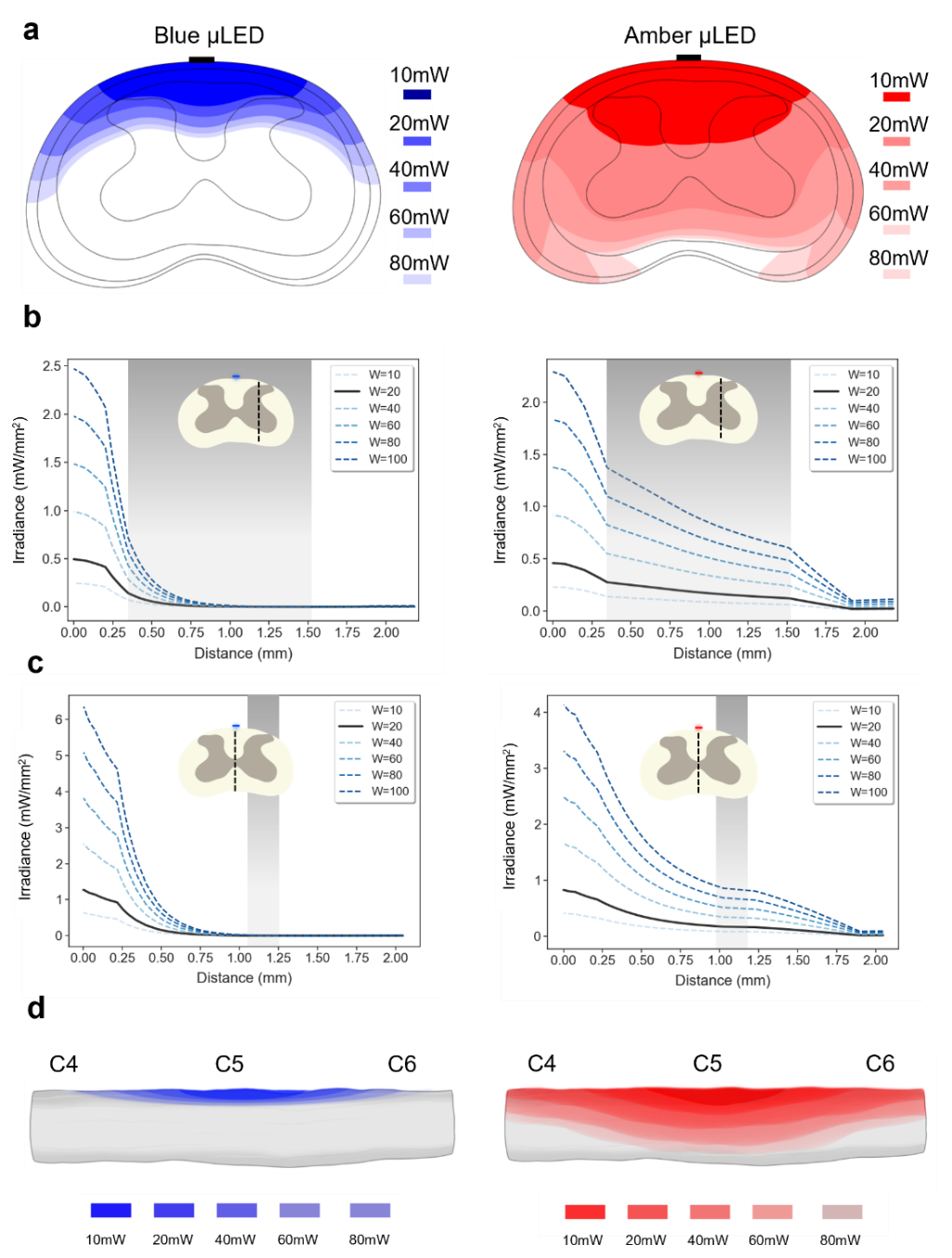
**

**Extended Data Fig. 6 | Optical characterization of the light-tissue interaction in rats. a**, Finite element simulations of irradiance distribution in a rat spinal cord for blue (470 nm, left) and amber (590 nm, right) wavelengths. Regions with magnitudes below the threshold of 0.1mW/mm² were excluded from the simulation results, and the μLED electrical input powers ranged from 10 mW to 80 mW. **b**,**c**, Quantitative dorsal–ventral intensity profiles at various electrical input powers for blue (left) and amber (right) lights, shown for two different cut lines. Grey shading marks the depth range corresponding to the spinal cord gray matter relative to the µLED. **d**, Side-view simulations of blue (left) and amber (right) lights from a µLED positioned at C5, illustrating emission spanning multiple spinal segments (C4–C6).

**
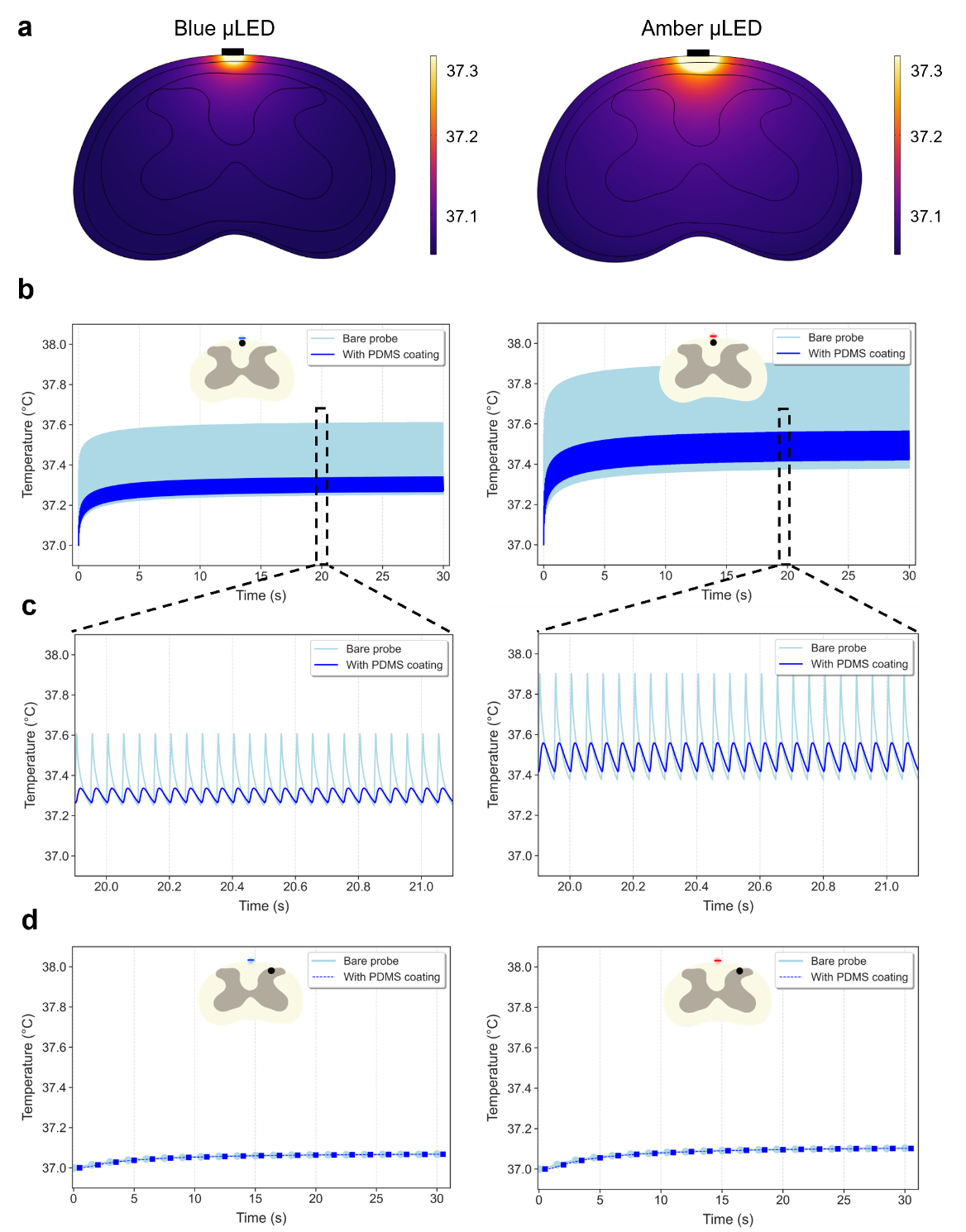
**

**Extended Data Fig. 7 | Thermal characterization of the light-tissue interaction in rats. a,** Simulated steady-state temperature distribution for blue (left) and amber (right) μLEDs after 30 s of continuous operation at 20 mW electrical input power. **b,c,** Temporal evolution of surface temperature at the probe–tissue interface for blue (left) and amber (right) μLEDs with parylene-C only and with additional PDMS encapsulation, shown across the initial 30 s (**b**) and for t = 20 s to t = 21 s (**c**). **d,** Simulated temperature change at the dorsal boundary of the grey matter for blue (left) and amber (right) μLEDs with or without PDMS encapsulation.

**
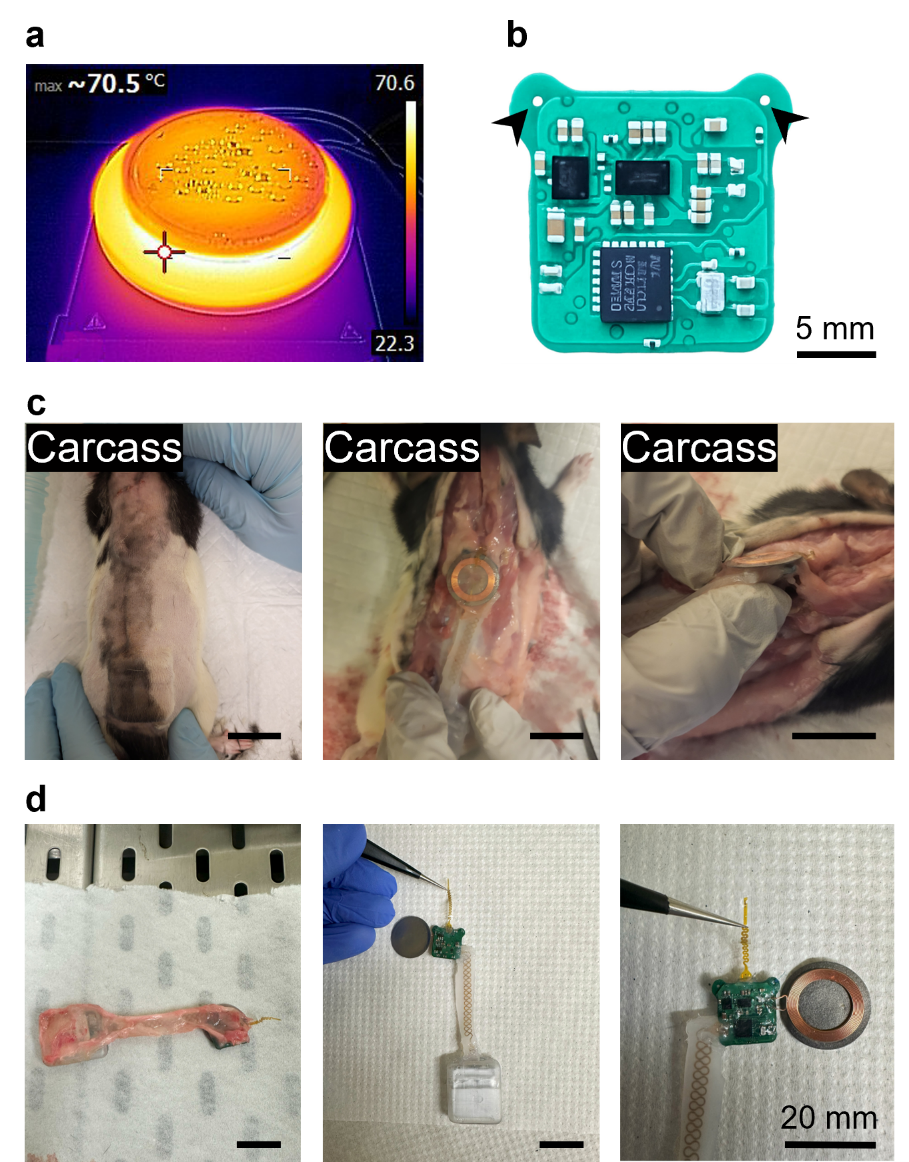
**

**Extended Data Fig. 8 | Optoelectronic device encapsulation durability and *in vivo* verification. a**, Accelerated aging test performed by submerging devices in PBS under stirring at 70°C. **b**, Optical image of the device platform highlighting peripheral suture holes (indicated by arrows). **c**,**d**, Gross examination following skin incision and tissue harvesting at the implantation site in rat carcasses (**c**), showing intact skin, muscle, battery, electronics, and interconnects, with a thin connective tissue layer encapsulating the device (**d**).

**
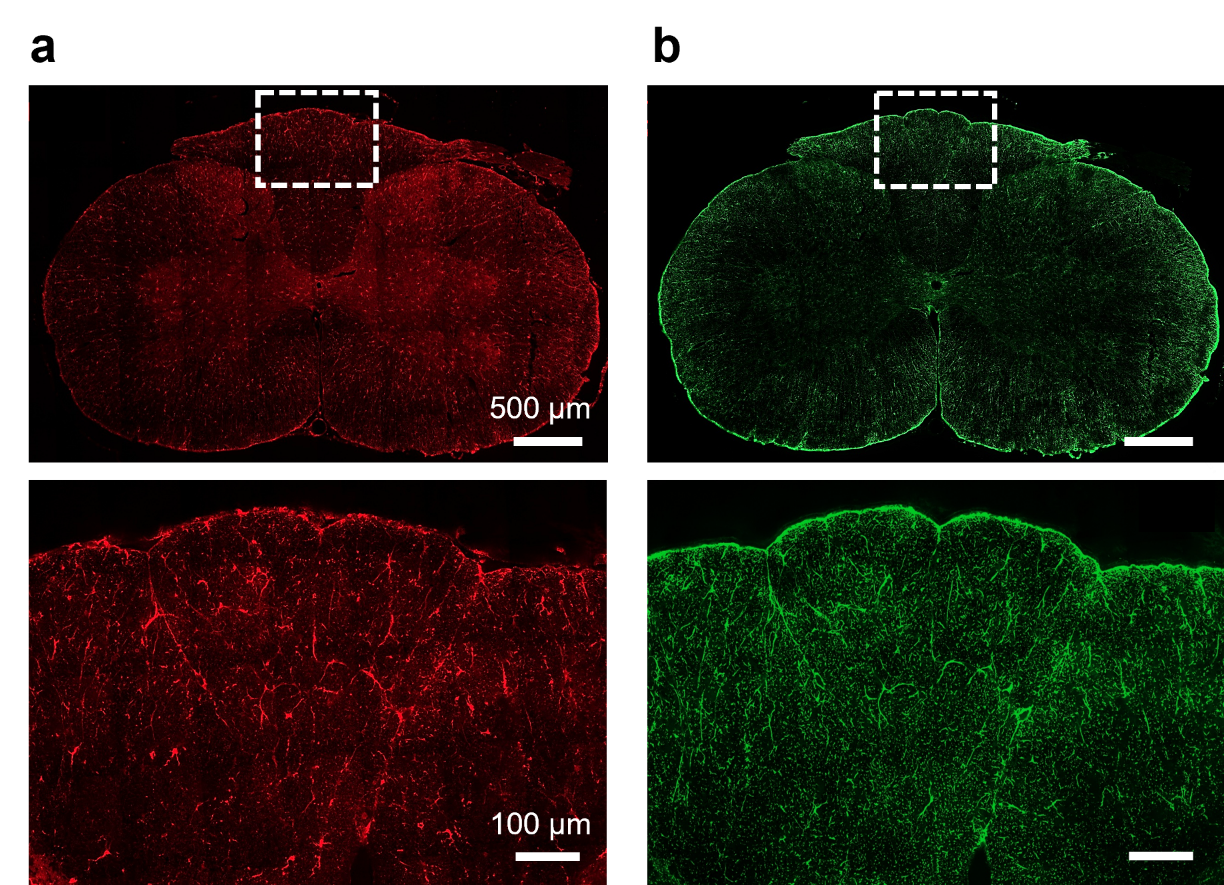
**

**Extended Data Fig. 9 | Immunohistochemical staining of the spinal cord for sham surgery in rats. a**,**b**, Representative immunohistochemical staining of the spinal cord at C5 from sham-surgery animals, showing expression of Iba1 in red (**a**) and GFAP in green (**b**).

**
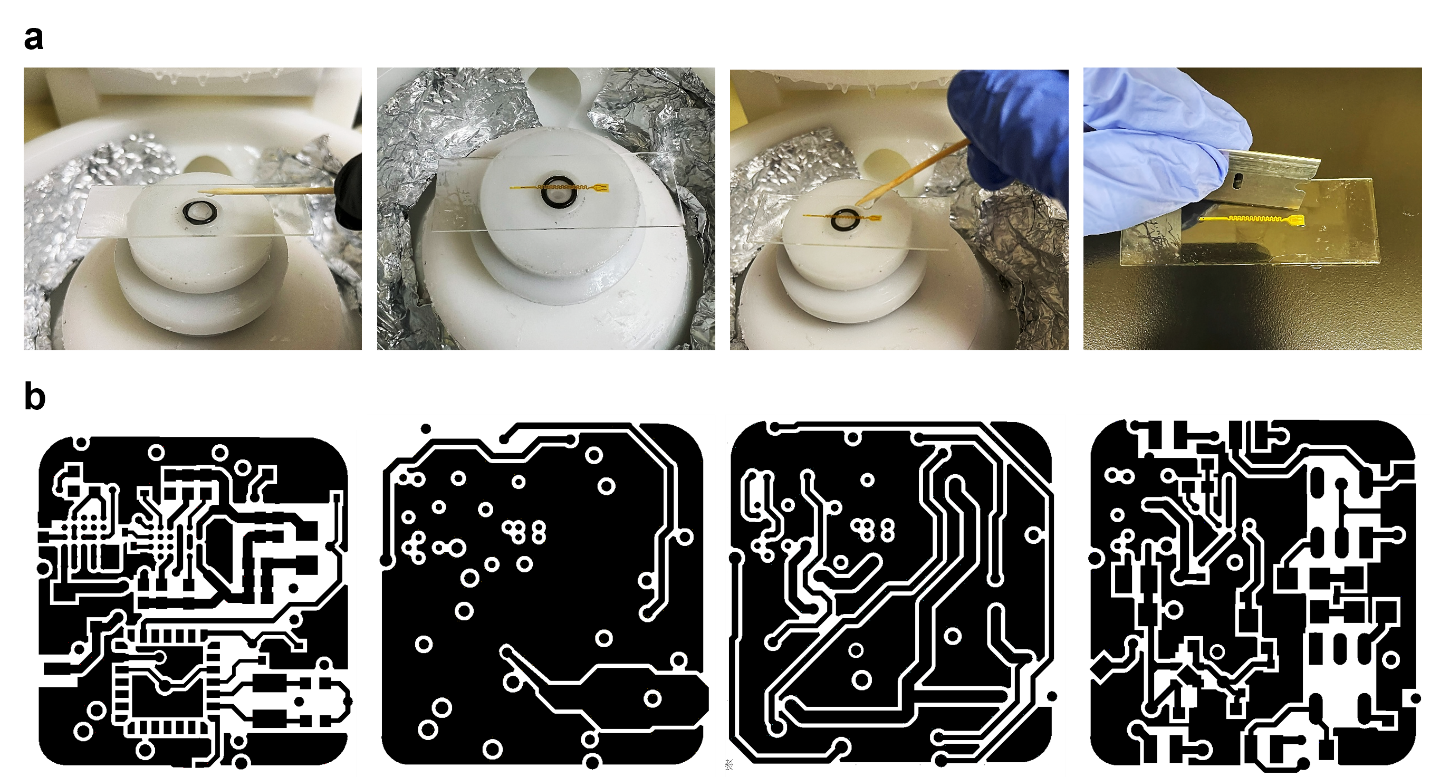
**

**Extended Data Fig. 10 | Micro-LED probes preparation steps and optoelectronic device electrical layout. a**, Stepwise encapsulation of µLED probes coated with parylene-C using PDMS. A base PDMS layer (20 µm) was spin-coated onto glass slides, cured, and followed by placement of the probe. A second PDMS layer (50 µm) was spin-coated to achieve full encapsulation, and extended curing completed the process. Probes were then cut to final dimensions and gently peeled from the substrate. **b**, Layout of the four-layer electronic board, incorporating an internal ground plane to enhance signal integrity and provide shielding from electromagnetic interference (EMI) during wireless charging of the device.

**Supplementary Note 1 | Wireless charging of the optoelectronic device after implantation**. Implanted devices could be wirelessly charged using any commercially available Qi-compatible chargers (Qi v1.2 or later). To enhance transmitter–receiver coil coupling, the charger housing was removed to directly expose the transmitter coil, which was then positioned above the subcutaneously implanted receiver antenna. A small permanent magnet was affixed to the center of the transmitter coil to facilitate precise coil alignment and allow stable attachment to the skin for continuous charging.

The wireless charger used in this study included a built-in LED indicator that provided real-time feedback on successful power transfer, remaining continuously on while power packets were transmitted and acknowledgement signals were received from the implanted device. In accordance with the Qi v1.2 protocol, the charger also incorporated a Foreign Object Detection (FOD) feature, which automatically halted wireless power transmission when metallic objects were detected between the transmitter and receiver coils to prevent overheating and unreliable, low-voltage buildup at the receiver. In such cases, the indicator LED blinked repeatedly to notify the user (Supplementary Video 7).

**Supplementary Note 2 | Horizontal ladder scoring.** The skilled horizontal ladder assay was performed following the standard protocol described by Metz and Whishaw^1^. For the missed steps analysis, the conventional 6-point scale was converted into two categories: steps rated **1-4** were classified as a Miss (score = 0), while steps rated **5 and 6** were classified as a Hit (score = 1). The detailed scoring criteria are summarized in the table below.

| Classification | Description |
| --- | --- |
| MISS | 0: A complete miss |
| MISS | 1: Deep slip, usually occurs when animal steps on a rung**,** but upon weight bearing a slip occurs |
| MISS | 2: Slight slip, slips after paw placement on a rung but does not disrupt walking |
| MISS | 3: Replacement, paw is moved to a rung quickly after placement, with no weight bearing taking place before replacing the position of the paw |
| MISS | 4: Correction, when a paw aims for one rung and is placed on another rung without touching the first rung. Also, when a paw is placed on a rung and then repositioned on the same rung before bearing weight. |
| HIT | 5: Partial Placement, when digits and heal/wrist is placed on a rung |
| HIT | 6: Correct placement, midpoint of the paw positioned directly on the rung while fully bearing weight |

**Supplementary Note 3 | Step Regularity Index (SRI)**. The CatWalk XT automated gait system defines the Step Regularity Index (SRI) as a measure of inter-limb coordination, quantifying how closely the stepping sequence during locomotion resembles a normal walking pattern. Higher SRI values (maximum of 1.0) reflect more coordinated and consistent gait patterns, whereas lower values indicate increased variability or disruption. All reported data was directly obtained from the CatWalk XT software.

**Supplementary Note 4 | Pre-implantation device preparation**. Prior to implantation, each device was carefully prepared and inspected to ensure smooth surfaces and uniform encapsulation. Particular attention must be given to rounding all device edges and reinforcing the corners with additional PDMS to minimize mechanical stress at the skin–device interface. Devices that did not meet this preparation criteria were excluded. Consistent with this preparation, rapid skin recovery was typically observed within 2–3 days post-implantation, and minor skin protrusion was observed in 2 mice out of 11.

**Supplementary Table 1 | Comparison of current battery‐free optogenetic tools with the proposed battery-powered system in this study**

| Work |  | Features and advantages | Limitations |
| --- | --- | --- | --- |
| Shin et al. (*2017)*^2^ |  | - Fully battery-free optical stimulation via inductive wireless power - Continuous optical output enabled without onboard energy storage | - Requires continuous RF field from a cage-mounted transmitter - Strong spatial non-uniformity in received power (edge vs center of cage) - Optical output is sensitive to animal position and coil orientation - No onboard control or programmability (stimulation tied to RF exposure) - Requires cleanroom/microfabrication facilities |
| Gutruf et al. *(2018)*^3^ |  | - Digital control of stimulation parameters (frequency, intensity) - Battery-free optical stimulation with improved orientation tolerance using dual-coil design for the transmitter | - Continuous RF power delivery is still required - Out-of-cage experiments remain impractical - Power transfer efficiency decreases outside optimized field regions |
| Zhang et al. *(2019)*^4^ |  | - Battery-free optical stimulation enabled by inductive wireless power - Unlimited operation time under continuous RF powering | - Optical stimulation depends on uninterrupted RF coupling - Power delivery varies with animal position and posture - Optical stimulation restricted to RF-powered environments - Requires cleanroom/microfabrication facilities |
| Yang et al. *(2021)*^5^ |  | - Battery-free optical stimulation with real-time wireless parameter control - Multiple independently addressable µLEDs | - Continuous RF powering required via cage-mounted coils - Requires a spatially confined RF power field - Performance degrades near enclosure boundaries - Requires cleanroom/microfabrication facilities |
| Ausra et al. *(2021)*^6^ |  | - Battery-free optical stimulation with expanded spatial RF coverage - Improved power uniformity through optimized antenna configurations | - Optical stimulation limited to a predefined RF-covered volume - Requires complex, application-specific transmitter design - Loss of optical output outside tuned regions |
| Ouyang et al. *(2023)*^7^ |  | - Battery-free optical stimulation supported by continuous RF power delivery - Closed-loop neuromodulation with sensing, processing, and stimulation | - Optical stimulation ceases if RF coupling is disrupted - Increased thermal and infrastructure constraints - Requires cleanroom/microfabrication facilities |
| Wu et al. *(2026)*^8^ |  | - Continuous optical output maintained through inductive powering - High-density (64-µLED) spatiotemporal stimulation patterns - Real-time wireless programmability | - Optical stimulation critically depends on uniform RF field distribution - Operation restricted to RF-powered environments |
| System presented in this work |  | - No reliance on continuous RF power transmission - **Stable and reproducible light output** independent of animal position, posture, or orientation - Enables optical stimulation **outside RF-powered enclosures**, supporting spatially unrestricted behavioral assays - Does not Requires cleanroom/microfabrication facilities - Optical stimulation powered by an **on-board implantable battery** | - Current implementation does not support real-time wireless reprogramming - Implantation is more complex than fully battery-free devices due to the inclusion of an on-board battery |

**References**

1. Metz, G. A. & Whishaw, I. Q. The Ladder Rung Walking Task: A Scoring System and its Practical Application. *J. Vis. Exp.* 1204 (2009) doi:10.3791/1204.

2. Shin, G. *et al.* Flexible Near-Field Wireless Optoelectronics as Subdermal Implants for Broad Applications in Optogenetics. *Neuron* **93**, 509-521.e3 (2017).

3. Gutruf, P. *et al.* Fully implantable optoelectronic systems for battery-free, multimodal operation in neuroscience research. *Nature Electronics 2018 1:12* **1**, 652–660 (2018).

4. Zhang, Y. *et al.* Battery-free, lightweight, injectable microsystem for in vivo wireless pharmacology and optogenetics. *Proc. Natl. Acad. Sci. U. S. A.* **116**, 21427–21437 (2019).

5. Yang, Y. *et al.* Wireless multilateral devices for optogenetic studies of individual and social behaviors. *Nature Neuroscience 2021 24:7* **24**, 1035–1045 (2021).

6. Ausra, J. *et al.* Wireless battery free fully implantable multimodal recording and neuromodulation tools for songbirds. *Nature Communications 2021 12:1* **12**, 1968- (2021).

7. Ouyang, W. *et al.* A wireless and battery-less implant for multimodal closed-loop neuromodulation in small animals. *Nature Biomedical Engineering 2023 7:10* **7**, 1252–1269 (2023).

8. Wu, M. *et al.* Patterned wireless transcranial optogenetics generates artificial perception. *Nature Neuroscience 2025 29:1* **29**, 234–245 (2025).
